# Assembly of Drosophila Centromeric Chromatin Proteins during Mitosis

**DOI:** 10.1371/journal.pgen.1002068

**Published:** 2011-05-12

**Authors:** Barbara G. Mellone, Kathryn J. Grive, Vladimir Shteyn, Sarion R. Bowers, Isaac Oderberg, Gary H. Karpen

**Affiliations:** 1 Department of Molecular and Cell Biology, University of Connecticut, Storrs, Connecticut, United States of America; 2 Department of Genome Dynamics, Lawrence Berkeley National Laboratory, Berkeley, California, United States of America; 3 Department of Molecular and Cell Biology, University of California Berkeley, Berkeley, California, United States of America; Fred Hutchinson Cancer Research Center, United States of America

## Abstract

Semi-conservative segregation of nucleosomes to sister chromatids during DNA replication creates gaps that must be filled by new nucleosome assembly. We analyzed the cell-cycle timing of centromeric chromatin assembly in Drosophila, which contains the H3 variant CID (CENP-A in humans), as well as CENP-C and CAL1, which are required for CID localization. Pulse-chase experiments show that CID and CENP-C levels decrease by 50% at each cell division, as predicted for semi-conservative segregation and inheritance, whereas CAL1 displays higher turnover. Quench-chase-pulse experiments demonstrate that there is a significant lag between replication and replenishment of centromeric chromatin. Surprisingly, new CID is recruited to centromeres in metaphase, by a mechanism that does not require an intact mitotic spindle, but does require proteasome activity. Interestingly, new CAL1 is recruited to centromeres before CID in prophase. Furthermore, CAL1, but not CENP-C, is found in complex with pre-nucleosomal CID. Finally, CENP-C displays yet a different pattern of incorporation, during both interphase and mitosis. The unusual timing of CID recruitment and unique dynamics of CAL1 identify a distinct centromere assembly pathway in Drosophila and suggest that CAL1 is a key regulator of centromere propagation.

## Author Summary

The centromere is essential for kinetochore formation, chromosome attachment to spindle microtubules, and equal segregation of the genome to daughter cells. Centromeres are epigenetically inherited through a unique type of chromatin which contains centromere-specific proteins. At each round of DNA replication, centromeric proteins become diluted and must be replenished to ensure faithful maintenance of the centromere locus through cell division. Whether divergent eukaryotes share a common strategy for centromere identity and propagation remains an unanswered question. Here, we examine how Drosophila centromere proteins re-distribute after replication, and we determine the cell-cycle dynamics of their replenishment. We show that three chromatin components required for centromere maintenance display distinct dynamics during the cell cycle; surprisingly, two components are assembled at centromeres during mitosis. These results suggest a new model for regulation of centromere assembly in Drosophila, which emphasizes a key role for the Dipteran-specific protein CAL1.

## Introduction

Centromeres are the chromosomal regions that mediate correct assembly of the kinetochore, a multi-protein structure necessary for attachment to spindle microtubules and faithful chromosome segregation in mitosis and meiosis. Centromeres are composed of DNA associated with nucleosomes that contain the H3 variant CENP-A (CID in Drosophila), and numerous constitutively bound centromeric proteins [1]. Specific underlying DNA sequences are neither necessary nor sufficient for centromere function in many eukaryotes, in contrast to the requirement for conserved, centromere-specific proteins such as CENP-A [1].

Accurate chromosome segregation also requires that the number and positions of centromeres be stably inherited through cell and organismal generations. DNA replication in mid to late S phase generates two copies of centromeric DNA [2], [3], but little is known about how passage of the replication fork affects the integrity of centromeric chromatin, how centromeric proteins are redistributed, and how intact centromeres are recreated after replication and accompanying nucleosome dilution. CENP-A assembly does not require DNA replication, in contrast to the replication-dependence of histone H3 deposition [2], [4]. Surprisingly, the timing of CENP-A replenishment during the cell cycle is not the same in different eukaryotes. In human HeLa cells, newly-synthesized CENP-A protein is recruited to centromeres during late telophase and G1, and requires mitotic exit [5]. GFP-CID and GFP-CENP-C recruitment in Drosophila syncytial embryos is initiated earlier in mitosis during anaphase. Interestingly, anaphase loading is not observed in later embryonic stages [6], where the cell cycle timing of loading has not been determined. GFP-CID was also previously reported to be deposited in G2 phase in Drosophila Kc167 cells [4]. What is conserved between Drosophila and human cells is that there is a delay between centromeric DNA replication (S phase) and CENP-A replenishment (mitosis or G1). Interestingly, this means that the main function of centromeres, *i.e.* kinetochore assembly and chromosome segregation in mitosis, occurs with only half of the maximal amount of CENP-A in these organisms [5]. In contrast, in organisms such as *S. pombe* [7]–, Arabidopsis [10], and Dictyostelium [11] the kinetochore is assembled on chromatin containing a full CENP-A complement, which has led to the proposal that mitotic and post-mitotic CENP-A recruitment may have been acquired more recently during evolution [11]. Finally, the signaling event(s) responsible for initiating centromere replenishment have not been identified in any of these organisms.

Despite differences in the timing of centromere replenishment in the cell cycle, both *S. pombe* and human cells contain homologous proteins that are essential for CENP-A assembly, specifically the Mis18 complexes and the CENP-A partner Scm3/HJURP [12]–[16]. The timing of CENP-A assembly in human cells approximately coincides with centromere localization of HJURP [12], [13]. The human Mis18 complex (which contains hMis18α, hMis18β and M18BP/hKNL2) is recruited at centromeres at the end of mitosis [12], [13], [17], [18], slightly before CENP-A and HJURP [13], [19] and has been proposed to ‘prime’ centromeres to receive new CENP-A [17]. Studies of the turnover of several constitutive centromere proteins indicated that CENP-C displays dynamic exchange in G1 and G2 [20]. The timing and mechanisms controlling the replenishment of additional constitutive centromeric components (*e.g.* the CCAN [21], [22]) in human cells are not known.

Functional screens and database searches have failed to identify hMis18, M18BP1 or Scm3/HJURP homologs in Drosophila [16], [18], [23], [24], so it is unclear whether non-homologous proteins perform analogous functions in this organism. Centromeric CID localization in Drosophila requires CENP-C, CAL1, Cyclin A and Rca1 [23], [24]. CID, CAL1 and CENP-C interact physically, and are interdependent for centromere localization. CAL1 is particularly intriguing because it has only been found in Dipteran species, displays unusual localization dynamics in mitosis [23], and appears to limit how much CID and CENP-C can be incorporated at centromeres [25].

The anaphase recruitment of CENP-A and -C observed in Drosophila syncytial embryos may not be representative for this organism, since these embryos undergo very fast (8–10 min) nuclear divisions that lack G1 and G2 phases. Furthermore, the distribution to daughter cells and turnover of CID, CAL1 and CENP-C have not been determined. Thus, establishing the cell cycle dynamics of nascent centromere protein assembly in cells with a complete cell cycle is crucial for determining the general mechanism of centromere replenishment in flies.

Here, we report the dynamics of centromere protein redistribution and replenishment in Drosophila tissue culture cells. The SNAP tag system [5] was used to track the behavior of CID, CAL1 and CENP-C in pulse-chase and quench-chase-pulse experiments. CID and CENP-C are stable proteins, whose centromeric levels decrease by 50% at each cell division, as predicted for semi-conservative segregation and inheritance of centromere components. In contrast, CAL1 is less stably associated with the centromere because its centromeric levels decrease 66% after one cell cycle. Tracking of newly-synthesized protein demonstrates that CID is recruited during metaphase in Drosophila tissue culture cells, prior to what was reported for early fly embryos and HeLa cells, while CENP-C is recruited during both mitosis and interphase. Interestingly, CAL1 is recruited at centromeres during prophase, before CID, and, similarly to HJURP and CENP-A, physically associates with pre-nucleosomal CID [13]. These findings establish the temporal events leading to centromere replenishment in Drosophila cultured cells, and provide evidence that CAL1 plays a crucial role in targeting CID to centromeres.

## Results

### CID and CENP-C, but not CAL1, are equally distributed to daughter cells

Faithful transmission of the centromere locus depends on re-assembly of centromeric components that have been either diluted or disrupted by centromeric DNA replication. Semi-conservative inheritance of CENP-A was previously demonstrated in HeLa cells [5]. To establish how CID, CENP-C and CAL1 are inherited during cell division, we generated clonal cell lines expressing SNAP-CID, SNAP-CAL1 or SNAP-CENP-C fusion proteins (see Materials and Methods). The three centromeric proteins were expressed from the Copia promoter at comparable levels to the endogenous proteins [23], exhibited the expected centromeric localization, and did not perturb normal chromosome segregation (data not shown). The functionality of SNAP-CID was confirmed by the observation that segregation and viability defects associated with RNAi depletion of endogenous CID were rescued by SNAP-CID expression (Figure S1).

**Figure 1:**
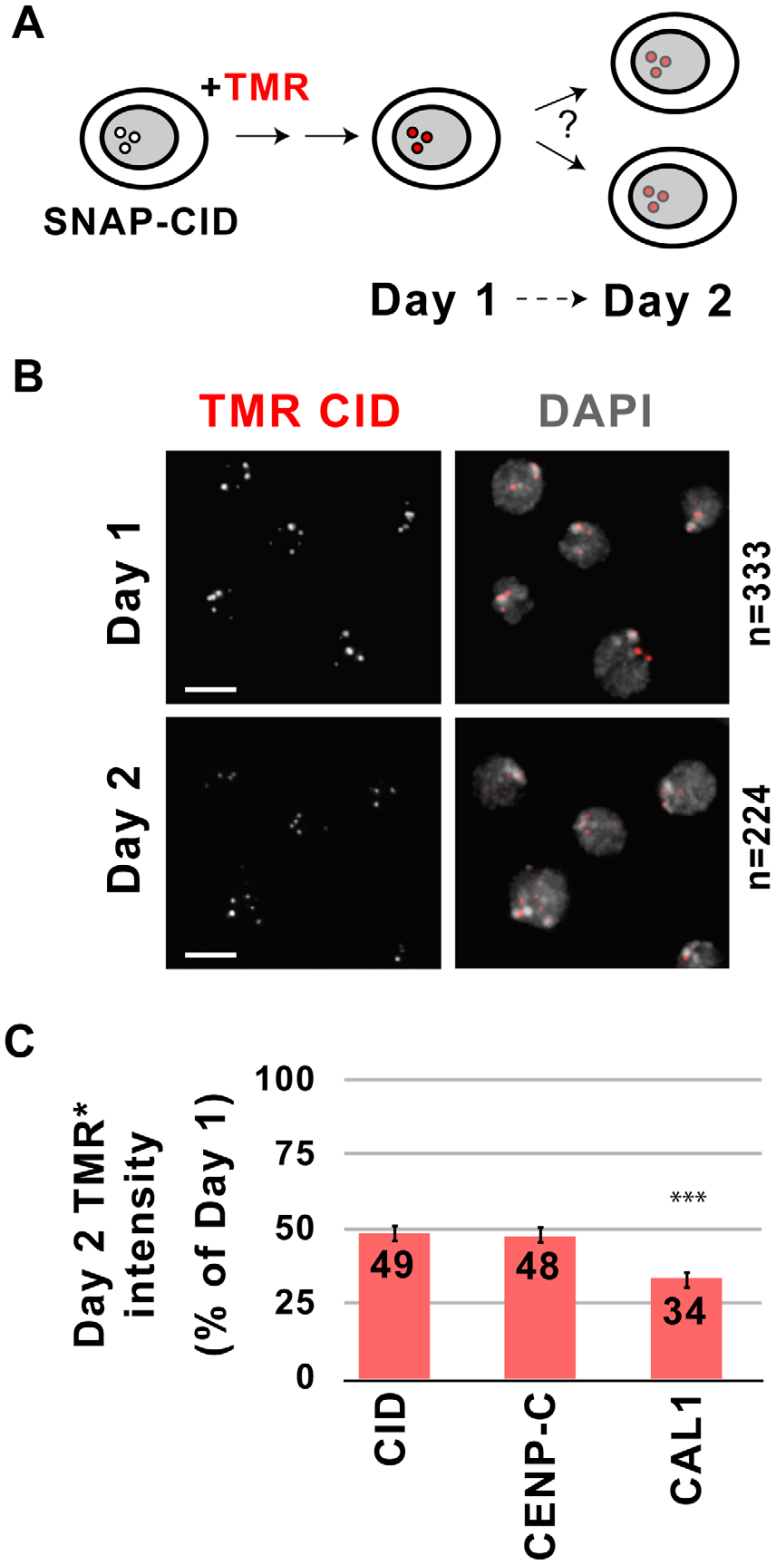
Inheritance of centromeric proteins through cell division. A) Experimental design for monitoring dilution of centromeric proteins during cell divisions. Clonal S2 SNAP-CID cells were incubated with TMR to fluorescently label CID. TMR-CID signal intensity was then quantified 24 h after labeling (Day 1) and 24 hours later (Day 2) to determine SNAP-CID distributions. B) Images from SNAP-CID cells imaged 24 h after TMR labeling (Day 1) and 24 hours later (Day 2). TMR-CID signal intensity (red) is reduced after one cell division. DAPI staining is shown in grey. n indicates the total number of cells quantified in two independent experiments. Bar = 5 µm. C) Quantification of the relative intensity of TMR-CID, CENP-C, and CAL1 at Day 2 on a per cell basis. The SNAP-tagged cell line of interest is shown on the x-axis, TMR intensity as relative percentage of intensities from Day 1 is shown on the y-axis. CID and CENP-C are distributed equally to daughter cells, while CAL1 displays significantly greater turnover during each cell division (*** p<0.0001, student's t-test). Bars represent standard error.

SNAP-tagged proteins were pulse-labeled using the cell permeable fluorescent substrate tetramethylrhodamine (TMR). TMR reacts with both centromeric SNAP and any SNAP from a soluble protein pool. Therefore, to determine the turnover of the pre-existing centromere pool, TMR signal intensity at centromeres was quantified 24 h after labeling (Day 1) and again after a 24 hr chase (*i.e.* after one doubling, Day 1, Figure 1A and Materials and Methods). This analysis showed that, on average, CID levels decreased to 49% after one cell division (Figure 1B, 1C). The distributions of intensity values for Day 1 and Day 2 demonstrate that the averages reflect the behavior of individual cells (Figure S2A). We conclude that pre-existing CID is distributed equally to daughter cells, as observed previously for CENP-A in HeLa cells [5]. These results also suggest that, on average, CID nucleosomes are segregated semi-conservatively to sister chromatids during DNA replication, but does not exclude conservative segregation of individual CID blocks.

The redistribution of pre-existing CAL1 and CENP-C during cell division could be different from that of CID, since neither appear to be nucleosome components [23], [25], [26]. Pulse-chase experiments using SNAP-CENP-C showed that the average TMR signal at Day 2 was 48% of the intensity at Day 1, indicating equal distribution of pre-existing CENP-C (Figure 1C, Figure S2B). However, TMR signal on Day 2 was only 34% for CAL1 (Figure 1C, Figure S2C), which is significantly lower than the 49% observed for CID (p<0.0001). Thus, 66% of pre-existing centromeric CAL1 is exchanged at each round of division. Similarly to CID, the distributions of intensity values for Day 1 and Day 2 for CENP-C and CAL1 are consistent with the averaged values (Figure S3A, S3B).

We conclude that pre-existing CID and CENP-C are stably retained at centromeres through cell division, and are diluted to levels consistent with 50∶50 segregation during replication in S phase. In contrast, CAL1 protein displays higher turnover (see Discussion).

### Newly synthesized CID is recruited to centromeres during mitosis

**Figure 2:**
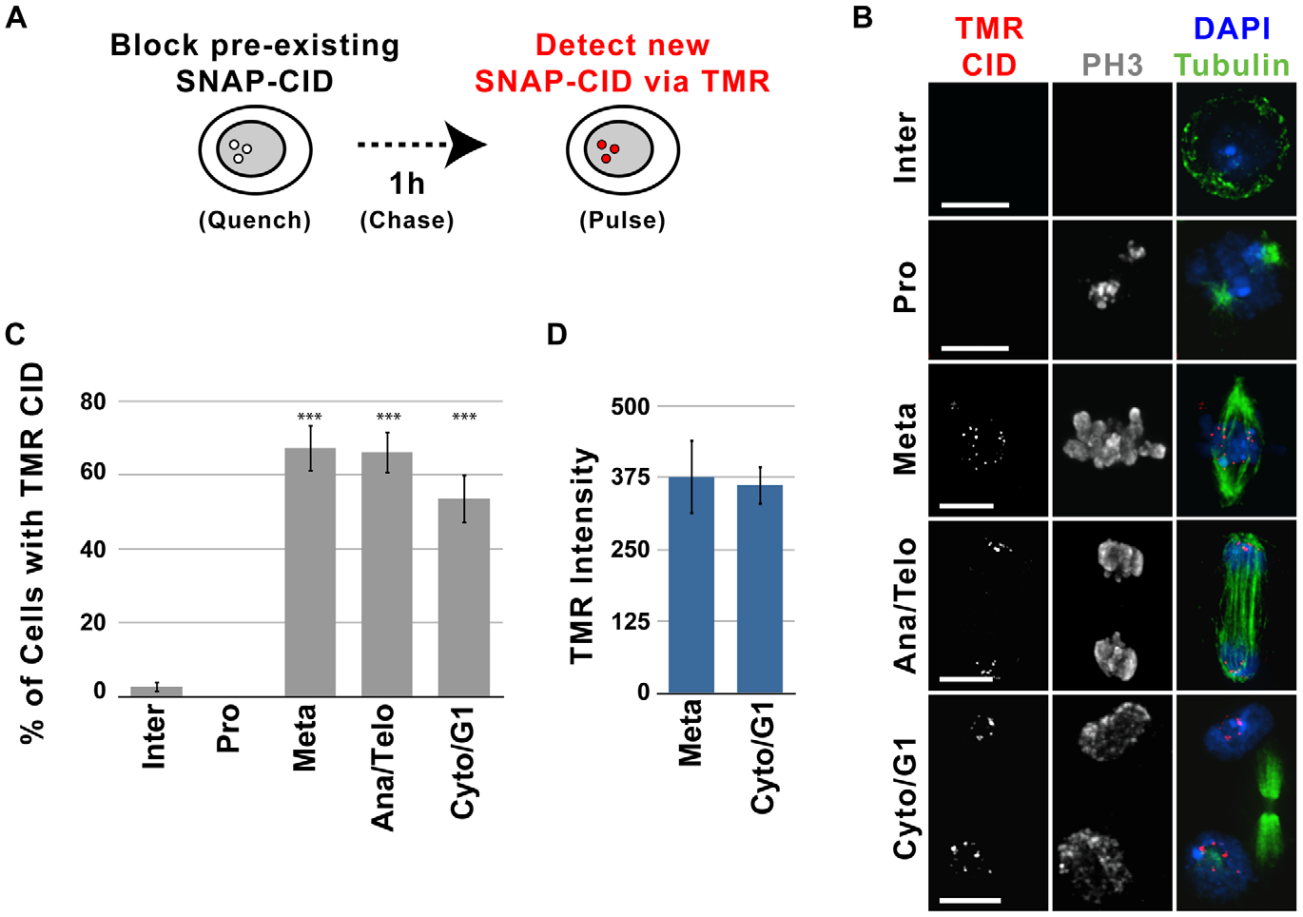
Recruitment of new CID occurs during metaphase in S2 cells. A) Experimental design for monitoring recruitment of new SNAP-CID during mitosis. Clonal S2 SNAP-CID cells were incubated with 12 µM BTP-block to render all pre-existing CID unable to react with TMR. After extensive washes, cells were harvested at different times following the block, and newly-synthesized CID was detected by incubation with TMR. B) Representative images showing recruitment of TMR-CID (red) 2 h after the BTP-block. Centromeric TMR-CID is rarely observed in interphase (Inter), but it is visible in metaphase (Meta), anaphase/telophase (Ana/Telo) and cytokinesis/G1 (Cyto/G1). IF with anti-tubulin (green), anti-phospho H3 (gray), and DAPI (blue) was used to identify specific mitotic stages. Bar = 5 µm. C) Average percent of interphase (n = 113), prophase (n = 37), metaphase (n = 55), anaphase/telophase (n = 29), and cytokinesis/G1 (n = 56) cells displaying centromeric TMR-CID 1 h following the BTP-block (X axis = cell cycle stage, Y axis = percent of cells positive for TMR labeling). New CID recruitment is visible within the first hour following the BTP-block in mitotic cells (*** p<0.0001 compared to interphase, Fisher's exact test). Data for different chase times are shown in Figure S2. D) Quantitative comparison of the TMR-CID intensity at centromeres in metaphase (n = 32) and cytokinesis, (n = 35). Cells in cytokinesis did not show a significant increase in TMR intensity (p = 0.5129, Mann-Whitney test), suggesting that new CID incorporation occurs mainly in metaphase. Bars represent standard error.

Faithful centromere propagation requires that new CID, CENP-C and CAL1 are recruited with each cell cycle. To determine the timing of deposition of centromeric proteins, we performed quench-chase-pulse experiments on clonal S2 cell lines expressing SNAP-tagged CID and GFP-tubulin (Figure 2A). Asynchronous cells were treated with the BTP-blocking agent to quench pre-existing SNAP-CID (Materials and Methods and Figure S4A), chased for 1, 2, 10 or 24 h to allow synthesis of new SNAP-CID, TMR labeled (pulse) and imaged (Figure 2A). All manipulations were carried out in conditioned medium (see Materials and Methods), and FACS analysis showed that cell cycle distributions remained constant throughout the experiment (Figure S4B).

S2 cells have a cell cycle that is approximately 24 h long, and newly-synthesized CID is already detected by TMR labeling 1 h after the BTP-block. To establish the approximate cell cycle stage(s) when cells first display centromeric TMR signal, cells were scored for the presence or absence of the mitotic-specific marker phosphorylated histone H3 Ser10 (phospho H3) [23]. Only 3–5% of S2 cells are in mitosis [27], and the length of mitosis is approximately 30 min [23]. Surprisingly, at 1 h after the BTP-block, 53% of cells in mitosis contained newly-synthesized SNAP-CID at centromeres (p<0.0001 compared to interphase), and 97.4% of all cells with centromeric TMR signal were in mitosis (Figure S4C). At later time-points after the BTP-block (2, 10, and 24 h), the percentage of mitotic cells that were TMR positive increased to 70%, 83% and 89%, respectively (Figure S4C), consistent with cells having more time to synthesize new CID protein before entering mitosis.

Although 95–97% of S2 cells are in interphase, only 2% of interphase cells contained TMR labeled CID at the 1 h time-point (Figure S4C). It is possible that some CID loading occurs in G1, S or G2 phases. However, since approximately 10% of S2 cells are in G1 (Figure S4A), loading exclusively in G1 would result in more than 2% TMR positive interphase cells. Furthermore, the efficiency of BTP-block was 97% in these experiments (see Materials and Methods), which could by itself account for the low frequency of interphase TMR-CID signals. In addition, 6–10% of cells complete mitosis in a 1 h interval after addition of BTP-block, and were scored as interphase in our quantitation. Although the percentage of TMR positive interphase cells increased at later time points, the frequencies were much lower than observed for mitotic cells (Figure S2C, p<0.0001 compared to mitosis for 2 h and 10 h), consistent with CID recruitment during the previous mitosis. Thus, although we cannot exclude that some CID incorporation occurs in G1, as observed in human cells, the vast majority must occur in mitosis. CID loading in mitosis was also observed in a clonal Kc167 cell line expressing SNAP-CID, demonstrating that these results are not specific to S2 cells (Figure S5).

To further assess the contribution of interphase to SNAP-CID loading, we quantified the number of interphase cells displaying TMR-CID in quench-chase-pulse experiments performed in S2 cells arrested with colchicine (see Materials and Methods). Colchicine disrupts microtubules and thus prevents cells from exiting mitosis and re-entering the cell cycle; thus, interphase cells displaying centromeric TMR-CID must have recruited SNAP-CID without going through mitosis. Clonal SNAP-CID cells were treated with colchicine for 2 h, BTP blocked and chased for 4 h in the presence of colchicine after which they were subjected to TMR labeling. We observed that 64% of interphase cells did not contain any centromeric TMR-CID, and the remaining 36% contained minimal TMR-CID signal compared to mitotic cells (Figure S6A). These results confirm that interphase contributes minimally to new CID loading and that the majority of interphase cells that were scored as TMR-CID positive in our time courses (Figure 2, Figure S4) were either non-BTP blocked cells or cells that re-entered the cell cycle after recruiting SNAP-CID in the previous mitosis.

We conclude that CID loading occurs predominantly in mitosis in S2 and Kc167 cells, and that minimal CID recruitment occurs during interphase, which distinguishes Drosophila from human cells, where the majority of CENP-A is recruited to centromeres in G1 [5].

### Centromeric recruitment of newly synthesized CID occurs in metaphase

To more precisely determine the specific stage(s) of mitosis when new CID is recruited, microtubules (GFP-tubulin), phospho H3 immunofluorescence (IF) and DNA morphology (DAPI) were used to identify cells in prophase, metaphase, anaphase, telophase and cytokinesis. The earliest mitotic stage where new CID was detectable was metaphase; 67% of metaphase cells and 0% of prophase cells were TMR positive 1 hr after the BTP-block (p<0.0001; Figure S6B, Figure 2B and 2C). Cells in anaphase, telophase and cytokinesis also displayed TMR-labeled CID at centromeres (Figure 2B). However, we observed that the total TMR intensity at centromeres did not increase between metaphase and cytokinesis (Figure 2D). Thus, the presence of newly-synthesized SNAP-CID in cells in anaphase/telophase and cytokinesis likely results from CID loading in the previous metaphase, and not from ongoing CID recruitment during later mitotic stages.

**Figure 3:**
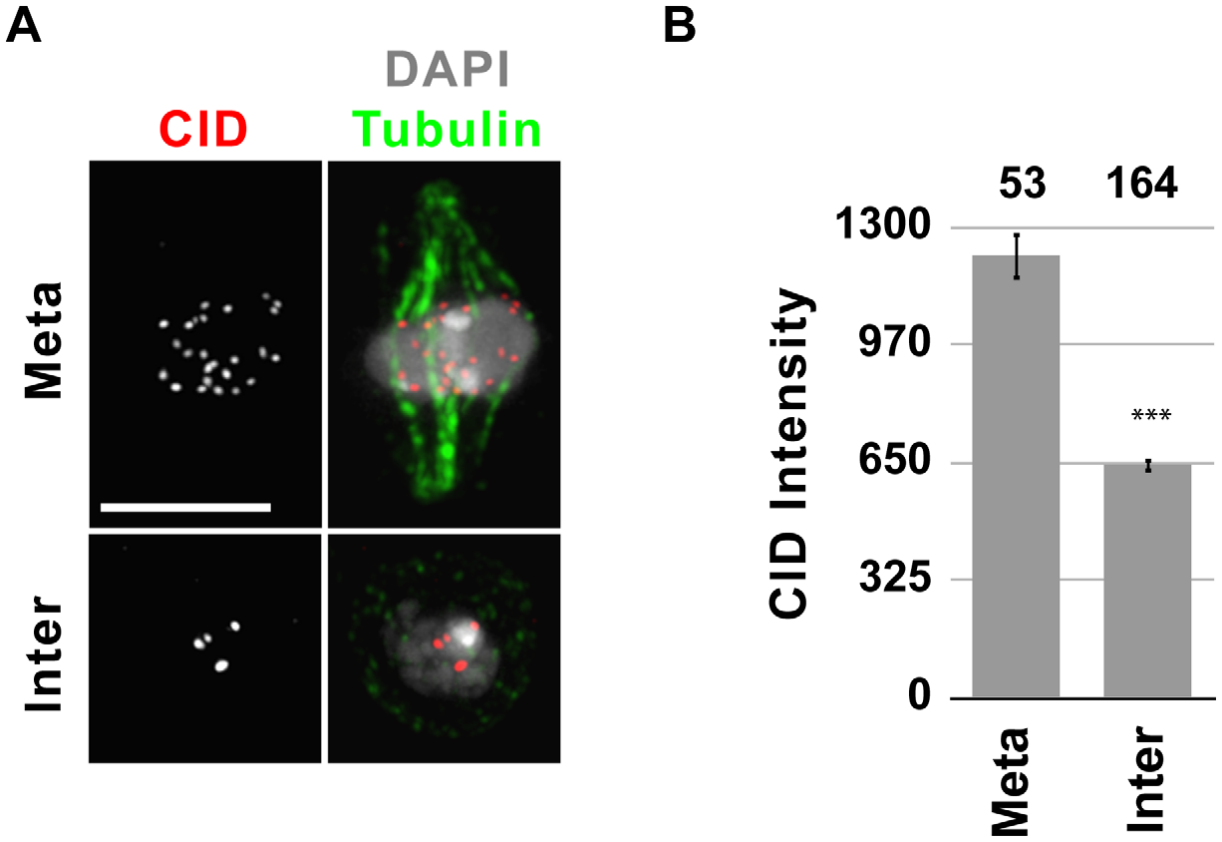
Metaphase cells contain double the amount of CID compared to interphase cells. A) Representative IF images of metaphase (meta) and an interphase (inter) cells stained with CID (red) and tubulin (green) antibodies, and DAPI (gray). Bar = 5 µm. B) Quantification of CID intensity. Total CID intensity was measured for individual cells and the mean CID intensity is shown in the graph. The number of cells quantified is shown above the graph. *** p<0.001 (paired T-test); error bars represent standard error.

To determine if endogenous CID is recruited to centromeres in metaphase, as observed for SNAP-tagged CID, we compared the total CID intensity in metaphase and interphase using IF in S2 cells (Figure 3). S2 cells have approximately 13 chromosomes whose centromeres form 3–6 distinguishable foci throughout interphase. De-clustering occurs in early mitosis, making individual centromeres distinguishable. Because of centromere clustering in interphase, each centromere focus is composed of several centromeres, making quantitative comparison of individual centromere IF signals between interphase and metaphase cells unfeasible. Therefore, we quantified the total nuclear CID intensity in interphase and metaphase cells (see Materials and Methods). If endogenous CID is recruited in metaphase, then G1, S and G2 cells should have similar total amounts of CID at centromeres, and metaphase cells should have double that amount. Indeed, the mean total CID intensity of metaphase cells was approximately 2-fold higher than in interphase (p<0.0001; Figure 3B), confirming that the cell cycle timing of endogenous CID recruitment is similar to that of SNAP-tagged CID.

In summary, our data show that newly-synthesized CID is assembled at centromeres during metaphase in S2 cells, and that recruitment occurs in a discrete ‘pulse’ during this stage. We cannot exclude the possibility that the process initiates slightly earlier, *e.g.* in G2 or prophase, and that centromeric CID needs to accumulate through metaphase to be detectable by SNAP-labeling and CID IF. Our experiments collectively show that CID assembly in Drosophila S2 cells occurs earlier in mitosis than observed in syncytial embryos (anaphase), and in human HeLa cells (late telophase through G1) [5], [6].

### Newly synthesized CAL1 is recruited to centromeres during prophase

**Figure 4:**
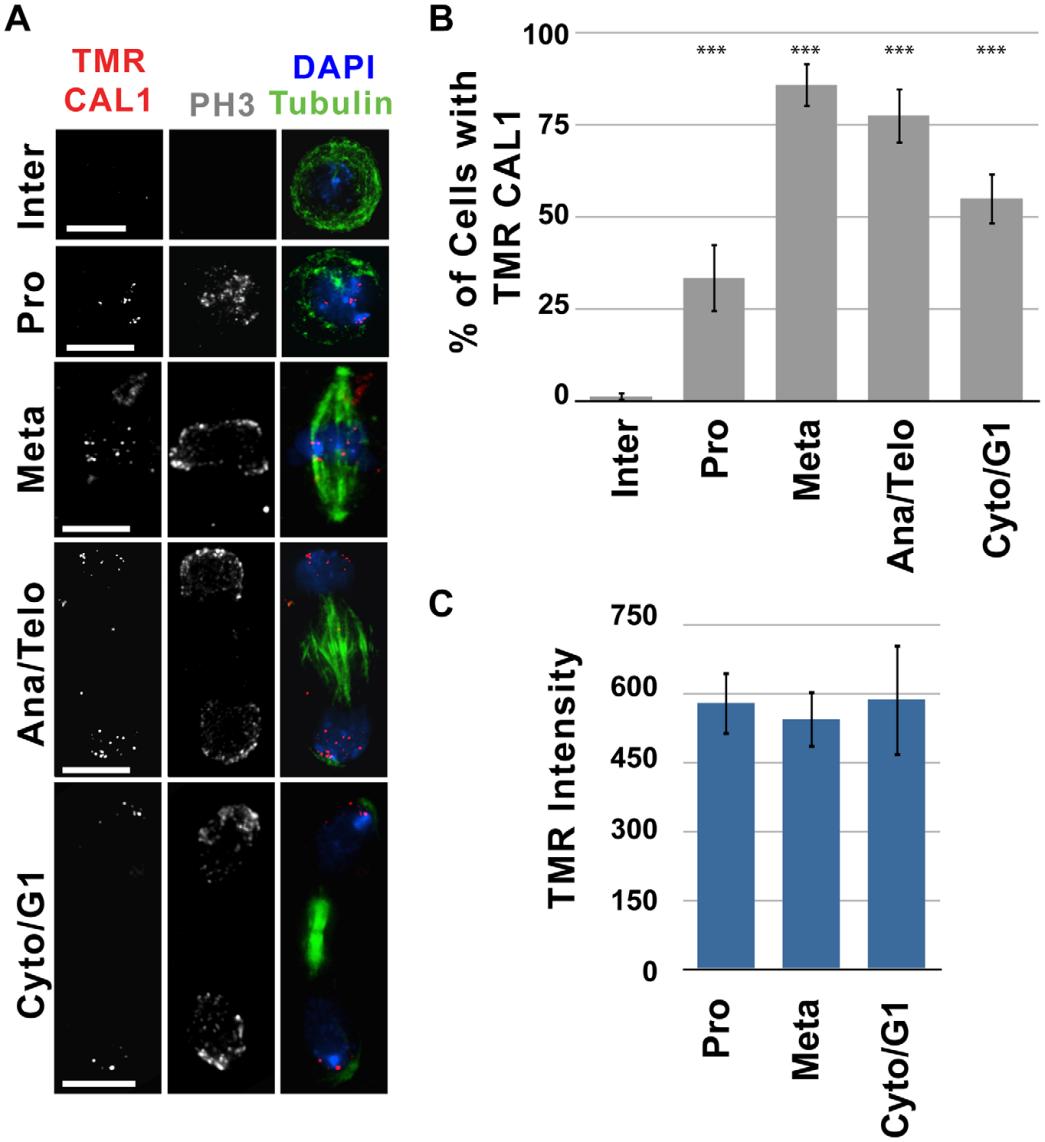
New CAL1 is first detectable during prophase in S2 cells. Quench-pulse-chase experiments were carried out as illustrated in Figure 2A. A) Representative images showing recruitment of newly-synthesized CAL1 (TMR, red) to centromeres 2 h after the BTP-block. TMR-CAL1 is absent in interphase (Inter), but is visible in prophase (Pro), anaphase/telophase (Ana/Telo) and cytokinesis/G1 (Cyto/G1). IF with anti-tubulin (green), anti-phospho H3 (gray), and DAPI (blue) was used to identify specific mitotic stages. Bar = 5 µm. B) Average percent of interphase (n = 84), prophase (n = 27), metaphase (n = 35), anaphase/telophase (n = 31) cytokinesis/G1 (n = 51) cells displaying centromeric TMR-CAL1 1 h following the BTP-block (X axis = cell cycle stage, Y axis = percent of cells positive for TMR labeling). New CAL1 recruitment is visible within the first hour following BTP-block in mitotic cells, and is first visible in prophase (*** p<0.0001 compared to interphase, Fisher's exact test). C) Quantitative comparison of the total TMR-CAL1 intensity at centromeres in prophase (n = 13) metaphase (n = 9) and cytokinesis, (n = 12). TMR intensity did not change significantly with mitotic progression (p = 0.9226, One-way ANOVA), suggesting that new CAL1 incorporation occurs mainly in prophase. Error bars represent standard error.

The timing of assembly for CID, CENP-C and CAL1 could differ, despite the fact that they physically interact and are interdependent for centromere localization [23], [25]. Furthermore, the timing of CENP-C centromeric recruitment has only been analyzed in early syncytial Drosophila embryos, which have unusual cell cycles [6]. The cell cycle timing of new CAL1 and CENP-C recruitment to centromeres was determined by quench-chase-pulse experiments, using clonal S2 cell lines expressing SNAP-CAL1 or SNAP-CENP-C. Similar to what was observed for CID, newly-synthesized CAL1 was visible at centromeres 1 h after the BTP-block, predominantly in mitotic cells; 63% of mitotic cells contained CAL1 TMR signal at centromeres compared to only 1% of interphase cells (p<0.0001 compared to interphase; Figure 4A, 4B, Figure S7). The vast majority of all cells with centromeric TMR signal were in mitosis (99%, Figure S7), since new CAL1 was only observed in 1% of interphase cells. This likely represents incomplete BTP blocking or CAL1 loading in the previous mitosis (see above).

In contrast to CID, analysis of specific stages of mitosis showed that new CAL1 protein was detected as early as prophase; 33% of prophase cells contain TMR-CAL1 1 h after the BTP-block (p<0.0001 compared to interphase; Figure 4A, 4B). Quantitative comparison between cells in prophase, metaphase and cytokinesis showed no significant change in TMR-CAL1 centromeric intensity at these different mitotic stages (Figure 4C). We conclude that new CAL1 is predominantly loaded in a single ‘pulse’ during prophase, prior to assembly of newly-synthesized CID in metaphase.

Newly-synthesized CENP-C was not observed until 10 h after the BTP block in quench-chase-pulse experiments, at which point 47% of interphase and 50% of mitotic cells (all stages of mitosis) were positive for TMR (Figure S8A, S8B). The longer time required to observe TMR signals for CENP-C, compared to CAL1 and CID, makes it difficult to distinguish CENP-C deposition in interphase from mitotic deposition in the previous cell cycle. In contrast, 10 h after BTP-block of pre-existing SNAP-CID and SNAP-CAL1, approximately twice as many mitotic cells display new CID and CAL1 relative to interphase cells (Figure S4C and Figure S7). These results suggest that CENP-C recruitment occurs in both interphase and mitosis, and that new CENP-C recruitment occurs from a pool of soluble CENP-C, which is blocked by BTP-treatment and therefore not visible by TMR labeling after short chases. Thus, the dynamics of CENP-C recruitment in Drosophila cultured cells are similar to those observed in human tissue culture cells [20], but differ from the dynamics observed in early Drosophila embryos, where CENP-C's recruitment was observed in anaphase [6].

### Recruitment of CID does not require spindle microtubules

**Figure 5:**
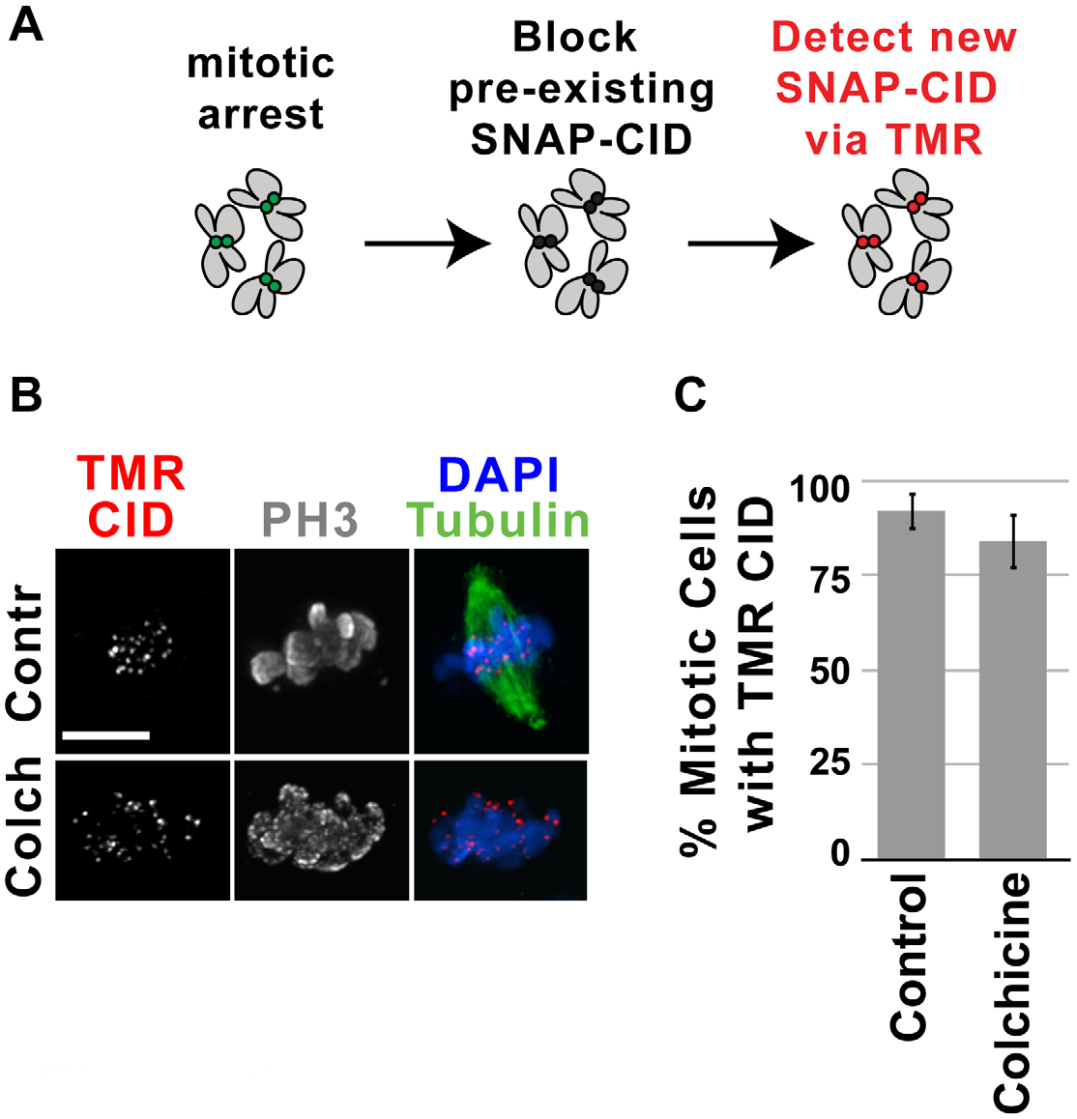
New CID recruitment occurs independently of microtubules. A) Experimental design for monitoring the recruitment of new CID during mitotic arrest induced by colchicine. Clonal S2 SNAP-CID cells were arrested in mitosis with either 12.5 µM colchicine or no drug (Contr) for two hours, then incubated with the BTP-block to quench all pre-existing SNAP-CID, and chased for four hours in the presence of colchicine. Newly-synthesized SNAP-CID was then detected by labeling with TMR. B) Representative images of SNAP-CID S2 treated (Colch) and not treated (Contr) with colchicine. TMR-labeled CID is shown in red, tubulin in green, phospho H3 in gray, DAPI in blue. Note that after treatment with colchicine cells lack spindle microtubules. New SNAP-CID recruitment occurs with efficiency comparable to untreated cells when cells are blocked in metaphase with colchicine (p = 1, Fisher's exact test). C) Control cells (no drug, n = 27) and cells treated with colchicine (n = 25) were scored for the presence or absence of centromeric TMR-labeled CID. Percent of cells (y-axis) with visible centromeric new SNAP-CID did not differ significantly (p = 0.4501, Fisher's exact test) in control cells compared to treated cells (x-axis). Error bars indicate standard error.

The observation that CID recruitment is restricted to metaphase raises the question of what signals and mechanisms regulate this process. It was previously proposed that robust kinetochore/microtubules interactions could provide a ‘signal’ for centromere replenishment [28], which would be consistent with CID loading during metaphase. We therefore examined whether new CID recruitment occurs in cells treated with the microtubule destabilizing drug colchicine. S2 cells have an intact spindle checkpoint [29] and respond to colchicine treatment by accumulating cells with condensed, phospho H3 positive chromosomes and no spindle microtubules. SNAP-CID cells expressing GFP-tubulin were incubated in the presence of colchicine, treated with BTP-block, chased and labeled with TMR to detect newly-synthesized CID (Figure 5A). We observed that 84% percent of the colchicine-arrested cells contained TMR CID, compared to 92% of untreated (control) cells (p = 1; Figure 5B). Thus, an intact mitotic spindle is not required for new CID recruitment in metaphase in S2 cells, similar to observations in HeLa cells and early Drosophila embryos [5], [6]. However, in those systems loading occurs after anaphase onset, so the spindle assembly checkpoint (SAC) had to be inactivated to assess CENP-A/CID assembly in the presence of microtubule disrupting drugs. Since CID is loaded during metaphase in S2 cells, we can conclude that CID recruitment occurs independently of intact mitotic spindles, mitotic checkpoint satisfaction and chromosome segregation. Furthermore, given that colchicine treatment abolishes inter-kinetochore tension, our results also exclude the contribution of tension and chromatin stretching in promoting new CID deposition [28].

### Proteasome activity and Cyclin A degradation are required for new CID loading in metaphase

**Figure 6:**
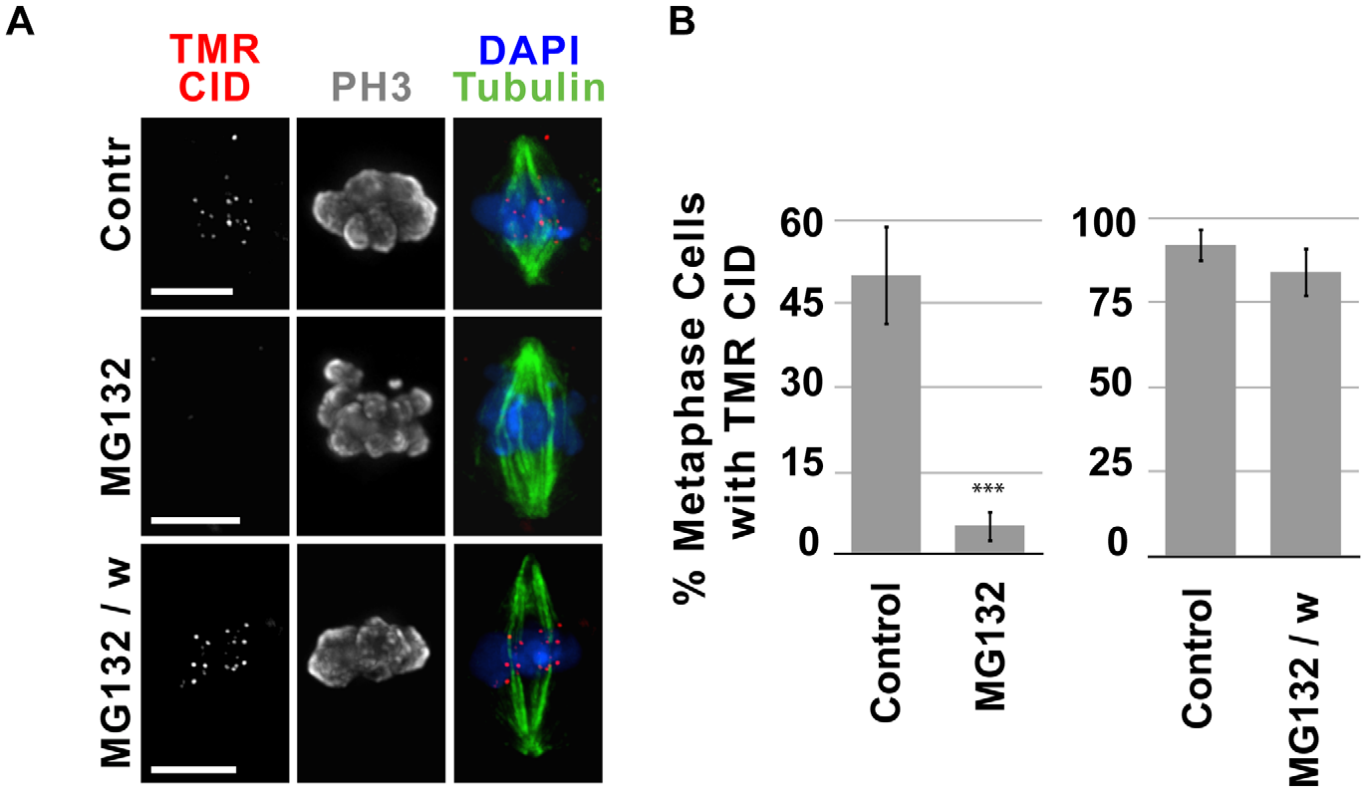
MG132 inhibition of the 26S proteasome suppresses recruitment of new CID. A) Representative images of untreated cells (Contr), cells treated with MG132 before and after BTP-block (MG132) and treated with MG132 prior to BTP block and washed with normal serum medium during chase (MG132/w). MG132 treatment blocked new CID loading, but this was reversible since removal of the drug by washing cells restores new CID loading. Tubulin is shown in green, phospho H3 in gray, TMR in red and DAPI in blue. Bar = 5 µm. B) In independent experiments, untreated cells (control, n = 29), cells treated with MG132 (n = 37; left panel) and untreated cells (control, n = 27) and MG132 treated cells followed by a chase without MG132 (n = 25; right panel) were scored for the presence or absence of centromeric TMR-labeled CID. The percent of cells (y-axis) with visible centromeric new SNAP-CID decreased dramatically in the presence of MG132 (p<0.0001, Fisher's exact test).

The observation that CID recruitment occurs independently of spindle function, checkpoint silencing and chromosome segregation, leaves open the question of what cell-cycle events regulate CID assembly in metaphase. Regulated ubiquitination of cyclins and other substrates followed by proteasome-mediated protein degradation are crucial to proper cell cycle progression, including the metaphase-anaphase transition [30]–[32]. Therefore, we analyzed whether proteasome inhibition by treatment with MG132 affects CID metaphase recruitment. SNAP-CID S2 cells were incubated for 2 h with 25 µM MG132, treated with BTP-block, chased for 4 h with or without MG132, and labeled with TMR. Treatment with MG132 did not affect the percentage of mitotic cells, however the percent of anaphases was significantly lower than in control cells (data not shown). While control cells efficiently recruited new SNAP-CID (55% of metaphases displayed TMR-CID; Figure 6A, 6B), treatment with MG132 dramatically prevented efficient recruitment (5% of metaphases displayed TMR-CID, p<0.0001; Figure 6). When the chase was carried out in the absence of MG132, new CID loading returned to levels comparable to control cells (Figure 6A, 6B). These results demonstrate that proteasome activity is crucial for efficient CID loading during metaphase, and suggest that one or more proteasome-targets must be degraded prior to or during metaphase in order to recruit new CID.

**Figure 7:**
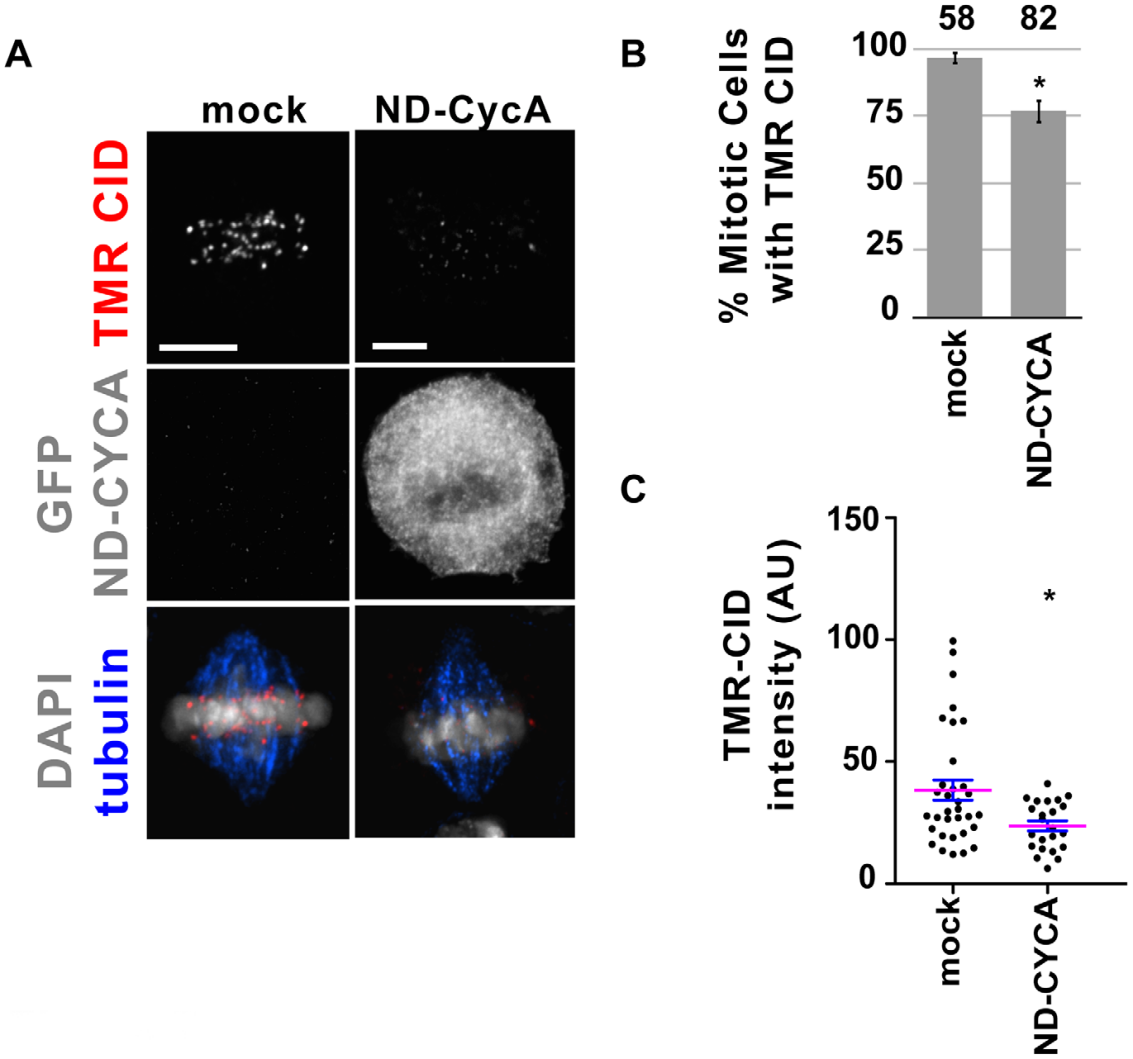
Presence of non-degradable CYCA in metaphase reduces the efficiency of CID recruitment. A) Representative images of metaphase cells transfected without plasmid (mock) and with GFP-Δ55-CYCA. After transfection, SNAP-CID S2 cells were BTP-blocked and TMR-labeled 4 h later. Cells transfected with GFP-Δ55-CYCA showed reduced SNAP-CID recruitment. TMR-CID is shown in red, GFP-Δ55-CYCA in gray, tubulin in blue and DAPI in gray. Bar = 5 µm. B) Percent of mitotic cells (y-axis) displaying centromeric TMR-CID 4 h following the BTP-block. Fewer cells recruited new CID when transfected with GFP-Δ55-CYCA (n = 82) compared to mock-transfected cells (n = 58) (* p<0.05; Fisher's exact test). Error bars represent standard error. C) Distribution of the average TMR-CID intensity in mock and transfected cells. Each dot represents individual cells. Mean (magenta) and standard error bars are shown (* p<0.05; Fisher's exact test). AU = arbitrary units.

One of the key events in metaphase is the Anaphase Promoting Complex (APC)-mediated ubiquitination of Cyclin A (CYCA) [30], which is then targeted for destruction by the proteasome [31], [32]. Cyclin A is centromere-associated and is required for CID localization [23]. Since MG132 inhibits new CID loading, we investigated whether CYCA degradation is required for new CID recruitment in metaphase, using a non-degradable CYCA mutant. S2 cells expressing SNAP-CID were transfected with a plasmid carrying CYCA lacking the destruction signals (ND-CYCA), fused to GFP [23], which cannot be degraded via the APC and causes a delay in metaphase and defective anaphases [33]. 24 h after transfection with ND-CYCA, cells were BTP-blocked, chased for 4 h and labeled with TMR to detect new SNAP-CID. Cells transfected with ND-CYCA showed a statistically significant decrease in the percent of cells in metaphase with new SNAP-CID compared to controls (77% versus 97%; p = 0.0011; Figure 7A, 7B). Furthermore, in cells transfected with ND-CYCA, the intensity of TMR-labeled SNAP-CID was significantly weaker in most cells (p = 0.0256; Figure 7A and 7C). We conclude that degradation of Cyclin A contributes to the process of new CID loading. The observation that MG132 treatment has a more dramatic effect suggests that CYCA is not the only proteasome-mediated degradation target involved in CID loading in metaphase. In summary, these observations demonstrate that proteasome-mediated degradation of CycA and other key targets is essential for centromere assembly in flies.

### CAL1 and CID are physically associated in chromatin-free extracts

**Figure 8:**
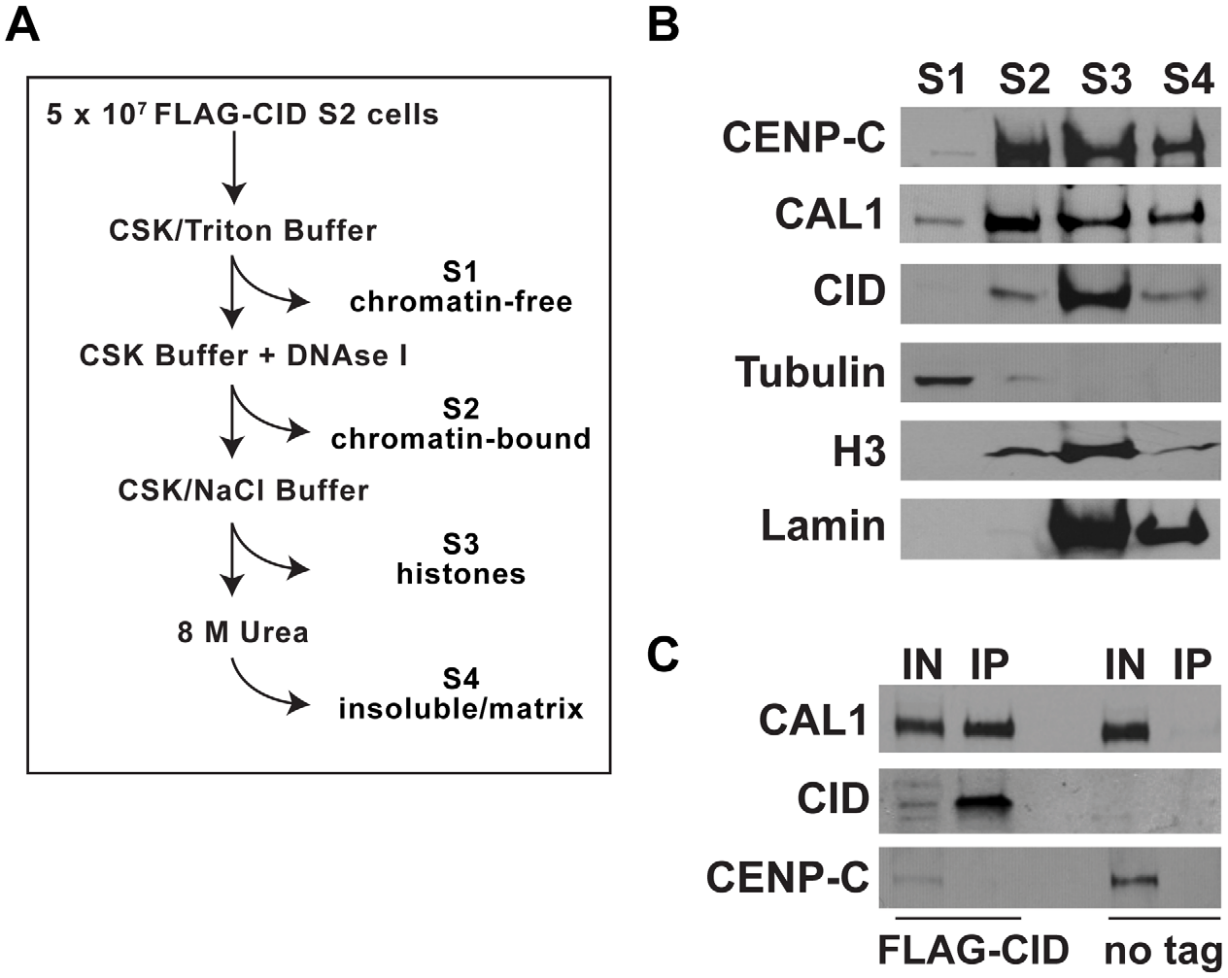
CAL1 and CID interact in chromatin-free extracts. A) Diagram depicting the cell fractionation method. B) Western blots to determine the presence of CAL1, CID and CENP-C in different cellular fractions. Chromatin-free (S1), nuclear-soluble (S2), chromatin-associated (S3) and insoluble/matrix-bound (S4) protein fractions were extracted from S2 cells stably transfected with FLAG-tagged CID. 40 µg of total protein was loaded in each lane. Antibodies against α-Tubulin, histone H3K4Me2 and Lamin were used to track the content of the different fractions (see Materials and Methods). C) Immunoprecipitation showing the specific interaction between FLAG-CID and CAL1 from the S1 fraction. The CID-CAL1 interaction was visible only in extracts from FLAG-CID expressing cells and not from untransfectd S2 cells (no tag). Soluble FLAG-CID did not pull down CENP-C. Chromatin-free extracts were incubated with 10 µl of FLAG-M2 agarose for two hours before elution of the bound CAL1 complex (IP) with 300 µg/ul of 3×FLAG peptide; 25% of the eluted material (IP) and 50 µg input material (IN) were loaded.

CAL1 displays functional similarities with two different sets of proteins required for CENP-A loading in human cells. CAL1 and components of the hMis18 complex are recruited to centromeres before CID and CENP-A, respectively, and exhibit similar dynamics in time-lapse analysis (loss from centromeres during mitosis [23]). However, hMis18 proteins do not bind CENP-A, whereas CAL1 physically associates with CID on chromatin and in yeast two hybrid assays [23], [25]. This property is instead shared with HJURP, which binds to human CENP-A in both chromatin and chromatin-free extracts [12], [13]. These observations led to the proposal that HJURP is a chaperone that facilitates targeting of new CENP-A assembly to centromeres [12], [13], [19], [34]. To determine if CAL1 also associates with pre-nucleosomal CID, we analyzed the distributions of CAL1, CID and CENP-C in different cellular fractions (chromatin-free (S1), chromatin-bound (S2), histone-containing (S3) and nuclear-matrix bound, insoluble material (S4)) prepared from S2 cell lines stably expressing FLAG-tagged CID (Figure 8A). The majority of CID and CENP-C protein were present in the S2, S3 and S4 fractions, whereas CAL1 protein was detectable in all four fractions, including chromatin-free extracts (S1) (Figure 8B). Immunoprecipitation with FLAG beads from chromatin-free extracts clearly identified the presence of CID (Figure 8C). Furthermore FLAG-CID specifically pulled down CAL1, indicating that FLAG-CID and CAL1 interact in pre-nucleosomal complexes (Figure 8C). In contrast, CENP-C was not present in these FLAG-CID precipitates, indicating that CENP-C association with CID and CAL1 is limited to chromatin-bound complexes (Figure 8C; [23], [25]). CAL1 is the first protein to be shown to bind to pre-nucleosomal CID in Drosophila. The finding that pre-nucleosomal CID interacts with CAL1 raises the possibility that CAL1 may be acting as a CID chaperone, targeting CID to centromeres at the appropriate cell cycle phase in a manner similar to HJURP (see Discussion).

## Discussion

We describe the cell cycle dynamics of three essential centromere components in Drosophila cells. CID, CAL1 and CENP-C display different turnover and assembly dynamics, despite the fact that these essential centromeric components interact physically, and are interdependent for centromere localization [23]–[25]. SNAP-tagged CID, CAL1 and CENP-C are expressed from the identical Copia promoter [23], thus it is unlikely that these distinctions are due to different rates of new protein synthesis. Using a pulse-chase strategy, we show that CID levels are reduced by ∼50% after one cell cycle, which could result from semi-conservative distribution of pre-existing CID nucleosomes, or random redistribution of parental CID-H4 tetramers [35], to replicated sister chromatids. While CID and CENP-C display stable association with centromeres and 50∶50 distribution after each cell cycle, 66% of TMR-CAL1 is replaced by new protein. Thus, CAL1 is either less stably bound, or its replenishment involves partial removal of pre-existing protein. Alternatively, CAL1 could undergo an even higher turnover and our quantification could be an underestimation; CAL1 could be entirely recruited *de novo* and the measured centromeric TMR-CAL1 could reflect recruitment from an initial soluble pool at the time of labeling.

An additional difference is that while SNAP-CID and CAL1 are detectable at centromeres 1 h after quenching the SNAP epitopes, 10 h of chase time are necessary for CENP-C to be visible by TMR labeling. This suggests that at each cell cycle the recruitment of CID and CAL1 relies for the most part on newly-synthesized protein, while CENP-C recruitment also involves a pre-existing non-centromeric or soluble pool. Indeed, the cellular fractionation analysis demonstrated the presence of low levels of CENP-C in chromatin-free extracts (Figure 8B), supporting the possibility that there is a soluble pool of CENP-C available to replenish the centromere-associated CENP-C diluted during the cell cycle.

CENP-C is targeted to centromeres during multiple cell cycle stages, consistent with previous findings in human cells [20]. In contrast, newly-synthesized CAL1 and CID are recruited to centromeres during discrete stages of mitosis. Using quench-chase-pulse time-courses in both asynchronous and arrested cultures, we demonstrate that the contribution of interphase to CID loading is minimal, since the percent of interphase cells displaying newly-synthesized SNAP-CID and the signal intensity of TMR-CID differ dramatically from those measured for mitotic cells (Figure 2 and Figure S6A). These observations distinguish Drosophila from human HeLa cells, where CENP-A is recruited during G1 [5], from fission yeast, where CENP-A assembles at centromeres in both S and G2 phases [7], [8] as well as from plants (G2) and Dictyostelium (G2/prophase) [10], [11].

**Figure 9:**
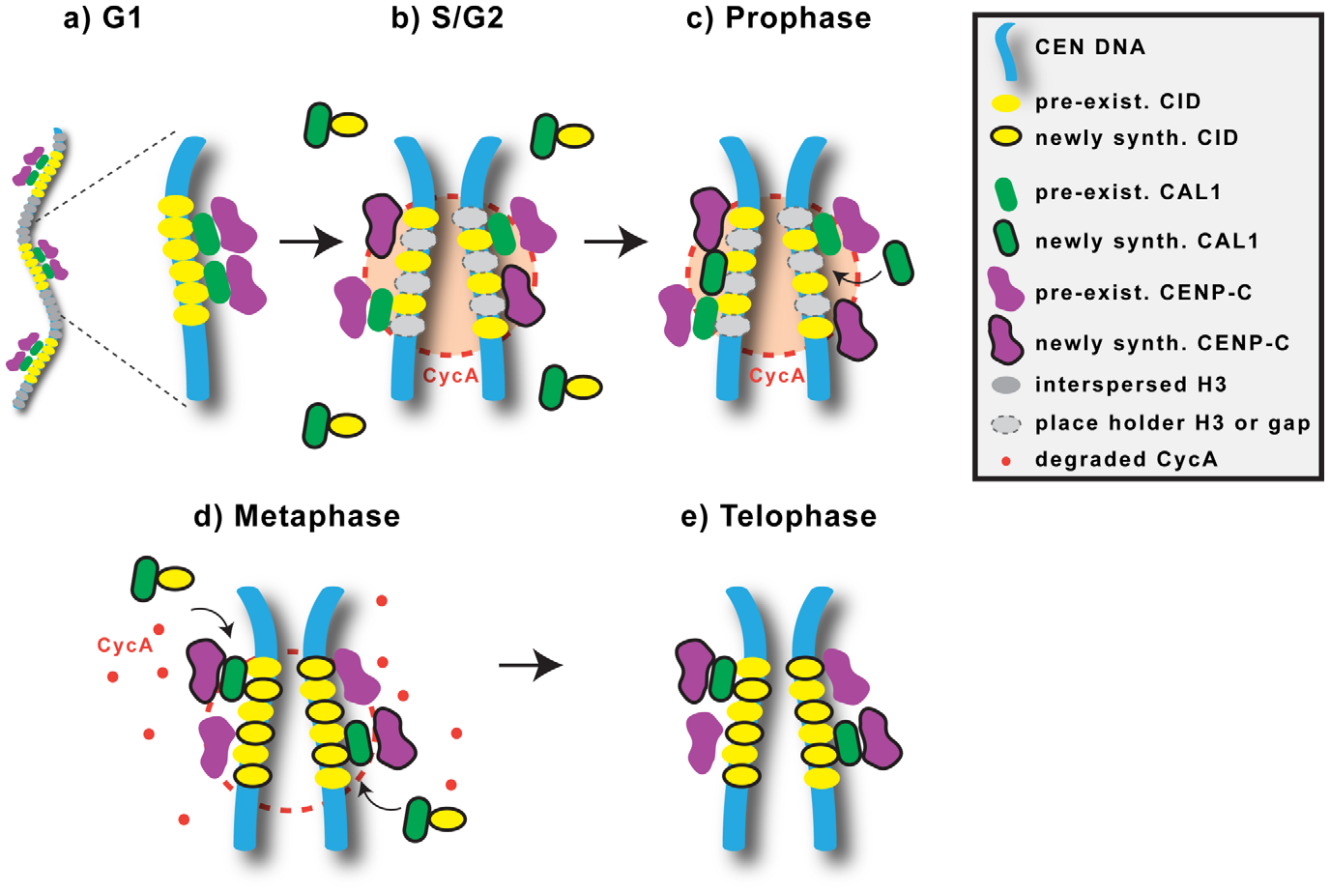
Model for centromere assembly in Drosophila cells. During the G1 phase of the cell cycle, the centromere contains the constitutive proteins CID, CENP-C and CAL1, which are reciprocally required for localization and interact physically. Blocks of CID nucleosomes (yellow) are interspersed with blocks of H3 nucleosomes (grey; not shown in zoom and subsequent phases for simplicity). Soluble CID is associated with CAL1. B) In S phase, centromeric DNA is replicated and, as a result, CID, CENP-C and CAL1 become diluted. Histone H3 may be deposited temporarily within CID blocks as a placeholder. Newly-synthesized CENP-C as well as pre-existing, soluble, CENP-C (not shown) are recruited to centromeres in interphase and mitosis (not shown). C) During prophase, free newly-synthesized CAL1 is deposited at centromeres, replenishing the diluted CAL1. CYCA is localized at centromeres throughout the cell cycle, and could prevent deposition of new CID until metaphase. D) During metaphase, CYCA is degraded, an event that promotes incorporation of new CID, pre-existing CAL1 dissociates from the centromere, and newly-synthesized CID is recruited at centromeres possibly by delivery through CAL1. E) In telophase, the levels of CAL1 increase again, by addition of pre-existing CAL1 at the sites left open by its dissociation during metaphase. Reduced levels of total CAL1 in metaphase may be required to ensure that the correct amount of CID is deposited [25].

Both new CID and CAL1 are assembled at centromeres in mitosis, but each protein is recruited during discrete stages: prophase for CAL1 and metaphase for CID. It is possible that CID and CAL1 loading are initiated simultaneously in prophase, but CAL1 levels accumulate faster than CID at centromeres. Regardless, the observed temporal distinction suggests that CAL1 acts upstream of CID recruitment (summarized in Figure 9). Incorporation of nascent CAL1 at centromeres during prophase could be mediated by binding to pre-existing centromeric CID and CENP-C. This could in turn promote new incorporation of nascent CID during metaphase, either by gap-filling or exchange of space-holder histone H3 (Figure 9).

Interestingly, a similar temporal distinction has been described for the human centromere proteins hMis18α, β and M18BP, which localize to centromeres in anaphase, before new CENP-A assembly in late telophase/G1 [5], [13], [17]–[19]. The lack of any physical interaction between hMis18α, β,M18BP and CENP-A [12], [13], [17], [21], and the observation that hMis18α can localize to centromeres even if CENP-A is depleted [17], has led to the proposal that this complex may ‘prime’ centromeres to receive new CENP-A [17] from the HJURP chaperone, whose centromeric targeting coincides temporally with deposition of new CENP-A [12], [13]. Homologs for hMis18 complex components and HJURP (or the budding and fission yeast Scm3 homologs) have not been identified in the Drosophila genome.

Collectively our data support a model in which CAL1 performs functions attributable to both HJURP and hMis18, despite the lack of sequence homology. hMis18 proteins are recruited to centromeres before CENP-A [5], [13], [17]–[19], and CAL1 loading precedes CID assembly. However, the hMis18 complex does not interact with CENP-A, whereas CAL1 and CID are associated in chromatin-free extracts, identifying the first Drosophila protein that binds CID in its pre-nucleosomal form. HJURP also interacts with pre-nucleosomal CENP-A [13], and both HJURP and CAL1 strongly colocalize with nucleoli [12], [23], [25]. Thus, CAL1 could ‘prime’ the centromere in prophase, and also mediate CID recruitment directly in metaphase (Figure 9).

We previously showed that gross-levels of centromeric GFP-CID and GFP-CENP-C did not visibly change through the cell cycle in time-lapse analysis [23], consistent with the 50∶50 segregation observed here during one division. In contrast, GFP-CAL1 levels were significantly reduced in metaphase, increased again in telophase, and remained stable through interphase [23]. The transient reduction in GFP-CAL1 levels at metaphase is intriguing, given that it coincides with new CID assembly. The observation that newly assembled TMR-CAL1 intensities were constant from prophase to cytokinesis (Figure 4C) suggests that most of the GFP-CAL1 reduction at metaphase and increase at telophase involves pre-existing protein. One model to account for these observations is that free CAL1 (not bound to CID) is recruited to centromeres in prophase where it performs a yet undefined ‘priming’ function; then, the subset of CAL1 bound to pre-nucleosomal CID escorts it to centromeres in metaphase while ‘old’ CAL1 is displaced (Figure 9). The interdependency of CAL1, CID and CENP-C in centromere localization [23] could be explained by the requirement of pre-existing CID and CENP-C for CAL1 assembly in prophase.

The loading of CID and CAL1 in specific, early stages of mitosis also raises questions about the nature of the signal(s) that initiate assembly of centromeric chromatin. Centromere replenishment signaling by kinetochore-microtubule interactions [28] is inconsistent with our demonstration that CID loading in metaphase is not affected by colchicine treatment, and therefore does not require spindles (as also observed in human cells [5]), SAC inactivation, chromosome segregation, or inter-kinetochore tension. However, we previously showed that premature activation of the Anaphase Promoting Complex, by Cyclin A or RCA1 depletion, interferes with CID localization to centromeres, demonstrating that centromeric chromatin assembly is linked to key regulators of mitotic progression [23]. Interestingly, Cyclin A localizes to centromeres and is degraded in metaphase [23], [36]; here we demonstrate that metaphase loading depends on proteasome activity, which could include degradation of key mitotic regulators. MG132 treatment prior to BTP block prevented CID loading (Figure 6) while transfecting cells with a non-degradable form of CYCA abrogated new CID recruitment in a subset of cells (23%, Figure 7B), and TMR-CID levels were significantly reduced in most cells. One possibility to explain the stronger impact of proteasome inhibition is that proteasome targets in addition to CYCA need to be degraded for efficient CID deposition. Alternatively, the presence of centromeric endogenous CYCA, which is probably degraded normally in the presence of excess ND-CYCA, might trigger a sufficient signal to initiate CID incorporation in some cells. Interestingly, Cyclin A is degraded in the presence of microtubule drugs and escapes inhibition of the APC by the SAC [37], which would explain why new CID recruitment takes place efficiently in the presence of colchicine (Figure 5). Proteasome and ubiquitin-ligase activities have been implicated in controlling proper CENP-A centromeric incorporation by degradation of euchromatic CENP-A in budding yeast and Drosophila [38]–[41]. Understanding the relationship between the CENP-A degradation pathway and our implication of proteasome activity in the recruitment of nascent CENP-A will require further investigation.

It is unclear at this point how degradation of CYCA contributes to CID assembly. One possibility is that high CDK-CYCA activity at the centromere inhibits CID recruitment, and that local inhibition of CDK activity through degradation of CYCA or other substrates triggers CID assembly. Understanding the role of degradation of Cyclin A and other APC and proteasome substrates in CID recruitment will be crucial to elucidating how centromere assembly is coupled to the cell cycle.

The dynamics of centromere replenishment in Drosophila cultured cells differs from those observed in *S. pombe* [7], [8] and human HeLa cells [5]. Early syncytial fly embryos display slightly later recruitment of new CID in anaphase, but this difference could be due to the unusually short nuclear cycles that lack G1 and G2 phases [6]. Although CENP-A loading in HeLa cells is first observed in telophase, it is possible that the primary signal to initiate CENP-A loading (*e.g*. inhibiting local CDK-CYCA activity at the centromere) is conserved, and occurs during prophase or metaphase in both Drosophila and human cells.

It is also puzzling that key proteins required in *trans* for CENP-A assembly, such as HJURP and CAL1, are not always conserved, in contrast to the universality of centromeric chromatin components such as CENP-A and CENP-C [1], [16], [18], [23], [24], [34]. It is possible that highly diverged proteins, such as CAL1, perform the same function(s) as human regulators such as HJURP and hMis18. Thus, although our data challenges the universality of centromere propagation dynamics in metazoans, it will be important to determine whether some mechanisms and signals required for CENP-A replenishment are conserved, despite different times of assembly in the cell cycle, and the lack of conservation for key regulatory proteins.

## Materials and Methods

### Generation of clonal S2 and Kc167 lines

S2 cells were co-transfected with either pCopia-SNAP-CID, SNAP-CAL1, or SNAP-CENP-C and pCoHygro (Invitrogen) and pAC-GFP-tubulin (gift of G. Goshima) using the Cellfectin reagent (Invitrogen). Polyclonal stable cell lines were generated by hygromycin selection. Clonal lines were generated as described in Zhang et al. (submitted). In brief, single cells were sorted into individual wells of a sterile 96-well plate using a DAKO-Cytomation MoFlo High Speed Sorter (UC Berkeley FACS Facility) containing 1000 untransfected S2 feeder cells in 200 µL of serum containing medium. After 1 week, the media was replaced with serum medium containing medium supplemented with hygromycin to establish monoclonal lines. Individual clonal lines were checked for expression and one line for each SNAP-tagged centromeric protein was used in our experiments.

To ensure that the SNAP-CID protein fusion is functional, RNAi was carried out using the soaking method as previously described [23], with double-stranded RNA with homology to the 3′UTR of the CID mRNA. These regions were amplified from genomic DNA by PCR. The primers contained the T7 promoter and were as follows: 3′UTR Forward TCCAAAAGAGAAGTTTAGG, Reverse CTCAATGACATGTTATTTATTTG. RNA was synthesized and precipitated using the Ambion kit following manufacturer's instruction, it was then denatured for 30 min at 65°C and re-annealed overnight. Cells were processed for IF with anti-CID (1∶1000), anti-tubulin (Sigma, 1∶500) and anti-SNAP (NEB; 1∶50) and imaged as described below.

### TMR labeling to track CID, CAL1, and CENP-C turnover at centromeres (pulse-chase)

In duplicate experiments, exponentially growing clonal S2 cells stably expressing SNAP-tagged centromeric proteins and GFP-tubulin were incubated in 300 µl of serum containing medium (SM) containing 4 µM tetramethylrhodamine (TMR) for 15 min. After 3 washes with 5 ml of SM, cells were counted, diluted to 1×10^6^ cells/ml and plated in a 12 well plate. Samples were taken, counted, fixed and mounted right after TMR (Day 0) and after 24 h (Day 1). Cell counting confirmed that cell numbers doubled during the 24 h period. Cells were imaged immediately after mounting and 10 fields of cells (200–300 cells) were acquired on a PersonalDV microscope (Applied Precision) using a 60×/1.42 Olympus oil immersion objective. Increments in z were set at 0.3 µm, sample thickness was 11 µm, and the bin was set to 2×2. 100× bin 1×1 images were also acquired to make the figures. All images were scaled in Softworks, maintaining the parameters constant between samples, saved as .psd files and figures were assembled in Adobe Illustrator.

### TMR labeling of newly synthesized CID, CAL1, and CENP-C (quench-chase-pulse)

Clonal S2 cells stably expressing SNAP-tagged CID, CAL1 and CENP-C and GFP-tubulin or clonal Kc167 cells expressing SNAP-CID were diluted at 1×10^6^ cells/ml in serum medium two days prior to experiments. In addition, extra flasks of cells were prepared at the same concentration two days prior to experiments so that conditioned medium (CM) could be harvested. 1 ml of cells were plated in 12-well culture plates and allowed to settle. Medium was removed and replaced with CM containing 12 µM bromothenylpteridine (BTP; BTP- block) to quench SNAP-tagged protein, then incubated for 30 min with gentle rocking. Cells were washed four times with 1 ml of serum medium and the last wash was incubated for 30 min. One well of cells was harvested prior to the 30 min wash for the 0 h time-point, to ensure adequate quenching of SNAP-tagged protein. All other samples were harvested at 1, 2, 10, and 24 h following the addition of BTP. Once harvested, cells were pelleted at 600 g for 5 min, and then resuspended in CM containing 4 µM TMR to label the newly-synthesized SNAP-tagged protein. Cells were allowed to incubate for 15 min with gentle rocking. Cells were washed four times with 1.5 ml of CM and the last wash was incubated for 30 min. Cells were pelleted, resuspended in 1× PBS, settled on a glass slide, and fixed with 3.7% formaldehyde in PBS-T (PBS with 0.1% Triton X-100) for 10 min. Slides were washed three times for 5 min in PBS-T, rocking, and then were blocked in 5% milk in PBS-T for 20 min. Slides were incubated with 30 µl of PBS-T 5% milk containing a polyclonal anti-phospho H3 antibody (Millipore; 1∶1000 dilution) for 2 hours at room temperature in a humid chamber. Slides were washed three times for 5 min in PBS-T, with gentle rocking, and then were incubated with Alexa 647 anti-rabbit antibody (Molecular Probes; 1∶500 dilution) for 45 min at room temperature in a humid chamber. Slides were washed three times for 5 min in PBS-T, with gentle rocking, and were then mounted on coverslips with SlowFade Gold Reagent (Invitrogen) containing 2.9 µM DAPI. Slides were imaged using a 60×/1.42 Olympus oil immersion objective on a PersonalDV microscope (Applied Precision) keeping exposure constant between all samples. Cells were manually scored for the cell cycle stage and for the presence or absence of centromeric TMR. Any daughter cell pair connected by a midbody was categorized as cytokinesis. More than 3 independent experiments were carried out, which showed similar results. At least 100 mitotic and 100 interphase cells were scored per experiment. Images were scaled in Softworks, maintaining the scaling constant between samples, saved as .psd files and figures were assembled in Adobe Illustrator. P-values were calculated in InStat (GraphPad).

### FACS analysis

5×10^5^ cells were centrifuged for 5 min at 600 g at room temperature and resuspended in 150 µl of PBS. 350 µl of ice-cold 100% 200 proof ethanol was added drop wise while vortexing cells gently. Cells were incubated at 4°C for 24 h, washed twice with 1 ml of PBS and then resuspended in 1 ml of PBS-T containing 20 µg/ml Propidium Iodide and 0.2 mg/ml RNAse A and incubated at 37°C for 15 min. Samples were analyzed on a Beckman-Coulter EPICS XL flow cytometer and the data was analyzed in FlowJo. Approximate percentage of cell in each cell cycle phase was estimated using the Watson-pragmatic model in Flowjo, eliminating doublets resulting from cells in cytokinesis/G1.

### Quench-chase-pulse with Colchicine and MG132 treatments

S2 cells stably expressing SNAP-tagged centromeric proteins and GFP-tubulin were diluted at 1×10^6^ cells/ml in serum medium two days prior to experiments and conditioned medium was prepared as above. 1 ml of cells was plated in duplicate wells of 12-well culture plates and allowed to settle. CM was prepared by harvesting medium from the additional flasks, filtering through a 0.22 µm filter, and diluting 1∶1 with serum medium. Once settled, cells were incubated for 2 h with either 12.5 µM colchicine or 25 µM MG132 in CM, washed three times with 1 ml CM, then incubated for 30 min with CM containing 12 µM BTP block followed by 3 washes in CM, the last wash being incubated for 30 min. One well of cells was harvested prior to the 30 min wash and treated with TMR as above for the 0 h time-point, where ∼50 metaphase cells were observed to ensure complete blocking of SNAP proteins (91% of interphase cells were efficiently blocked by treatment with BTP in these experiments). After BTP-block, samples were incubated in the presence of 12.5 µM colchicine or 25 µM MG132 for additional 4 h to allow synthesis of new SNAP-CID protein. Cells were then TMR labeled, fixed, stained with anti-phospho H3 antibody, mounted and imaged as described above. Presence or absence of TMR labeled centromeres was scored manually in two independent experiments (N = 50). Presence or absence of TMR-CID was also scored in interphase cells (phospho-H3 negative; N = 230) in the colchicine quench-chase-pulse (Figure S4). P-values were calculated in InStat (GraphPad).

### Quench-chase-pulse after transfection with ND-CYCA

Stable S2 cells expressing SNAP-CID were transfected with FUGENE (Roche) following the manufacturer's instructions with 2 µg of the pCopia-GFP-Δ55-CYCA plasmid, which was previously described [23]. 24 h post-transfection, cells were BTP-blocked as above, chased for 4 h and then incubated with TMR as described. Efficiency of the BTP-block was determined in both transfected (n = 66) and mock-transfected (n = 288) cells and was found to be 93% and 98% efficient, respectively. Imaging and manual scoring was carried out as described above. To determine the intensity of TMR-CID, the sum of of pixel intensity in the different z sections was averaged between 3 centromeres in each metaphase cell, the values obtained were subdivided in 5 groups (n = 35 ND-CYCA transfected metaphases; n = 22 mock-transfected metaphases). P-values were calculated in InStat (GraphPad) and the graph in Figure 5C was made in Prism (GraphPad).

### Quantification of CID, CAL1, and CENP-C turnover

In Softworx Suite, images were deconvolved with the method set to enhanced ratio, the number of cycles set to 5, and noise filtering set to medium. The images were then quick projected with the method set to max intensity. The images were exported as TIFF files, without scaling to min/max/exp values, with the destination computer set as Windows PC/Linux, and the output size set as 16-bit grey. The TIFF files were analyzed using a MATLAB (R2007a) script designed to measure total fluorescence intensity of TMR spots within a cell nucleus, the total area of those TMR spots and the median pixel intensity in a region within the nucleus but excluding the TMR spots. These data were exported as a text file and imported into a Microsoft Excel document. The true TMR intensity per cell was calculated by subtracting from the total TMR intensity the product of the TMR spot area and median pixel intensity of the nuclear region outside the spots. Statistical outliers were removed using the 1.5*IQR method. The remaining values were averaged across cells and within each day to make the comparison between days. The values for each experiment were normalized to Day 0, and then the resulting value was averaged between the two experiments. P-values were calculated using Student's t-test.

### Quantification of TMR-CID, endogenous CID, and TMR-CAL1 intensity in prophase, metaphase, and cytokinesis

S2 cells stably expressing SNAP-tagged CID or CAL1 and GFP-tubulin were blocked with BTP as described for the quench-chase-pulse experiments above. Samples were taken at 0 h and 4 h, labeled with TMR, washed and fixed as previously described. Cells were then stained with anti-phospho H3 antibody (Millipore; 1∶1000 dilution) for 2 hours at room temperature followed by staining with Alexa 647 anti-rabbit antibody (Molecular Probes; 1∶500 dilution) for 45 min at room temperature. Slides were mounted on coverslips using 30 µl of SlowFade Gold Reagent (Invitrogen) containing 2.9 µM DAPI and imaged on a Deltavision microscope as described above.

To compare the TMR intensity between cells in different mitotic stages between 9–13 cells per stage from two independent experiments were analyzed. The images were deconvolved with Softworx (in the “Ratio” mode, with 5 iterations) and quick projected. Using the 2D Model function, polygons were generated for individual cells in the DAPI channel to contain the entire DAPI area and the polygons were then propagated through the TMR (TRITC) or CID channel. The true TMR intensity (or CID intensity) per cell was calculated by subtracting the background for the TMR channel from the total TMR intensity within the DAPI mask. For the cells undergoing cytokinesis, the TMR intensity value for that image is the sum of the TMR intensity values for both daughter cells. Fisher's exact test (TMR-CID intensity in metaphase versus cytokinesis), one-way ANOVA (TMR-CAL1 in prophase, metaphase and cytokinesis), and Mann-Whitney Test (total CID intensity in metaphase versus interphase cells) were used to determine the p-values using InStat (GraphPad).

### Cellular fractionation and immunoprecipitation

5×10^7^ S2 cells stably expressing FLAG-tagged CID (where CID is expressed as a N-terminal fusion with FLAG under the pCopia promoter [23]) were washed in PBS before resuspension in CSK/Triton buffer (10 mM PIPES pH6.8; 100 mM NaCl; 1 mM EGTA; 300 mM Sucrose; 3 mM MgCl_2_; 1 mM DTT; 0.5% Triton-X100; 1× EDTA-free protease inhibitor (Roche); 1 mM PMSF) to extract the cytoplasmic and soluble nuclear fraction (S1). The remaining pellet was washed in CSK/Triton buffer and then resuspended in CSK buffer (without Triton-X100) with the addition of 25 U of RNase-free DNaseI (Promega) before incubation at 37°C for 30 minutes. 4M (NH_4_)_2_SO_4_ was added to a final concentration of 250 mM to disrupt the nuclear membrane and extract the chromatin-bound fraction (S2). After centrifugation the pellet was washed in CSK buffer before resuspension in CSK/NaCl buffer (CSK buffer+2M NaCl) to extract the histone-containing fraction (S3). The remaining insoluble fraction containing nuclear-matrix bound material, along with any precipitated proteins, was washed twice in CSK/NaCl buffer and then resuspended in 8M urea (S4).

Total protein concentrations in the four fractions (S1–4) were determined using the 660 nm Protein Assay (Pierce) and subsequently 40 µg of total protein were used for analysis by Western blot . Western blot with α-Tubulin antibodies (1∶1000, Sigma) was used to verify the extraction of soluble proteins (S1), histone H3 antibodies (H3K4 dimethylated, 1∶1000, Abcam) confirmed the extraction of nuclear soluble proteins (S2) and chromatin-associated (S3) fractions, lamin antibodies (1∶1000, Hybridoma bank) was used to follow the chromatin-insoluble/matrix-associated fractions S3–S4. FLAG-CID was detected using anti-FLAG antibodies (Sigma); CENP-C was detected with using affinity purified guinea-pig antibodies [23]; CAL1 was detected using affinity purified rabbit polyclonal antibodies (gift of Aaron Straight [23]).

The cytoplasmic and soluble nuclear S1 fraction was added to 10 µl of anti-FLAG M2 agarose (Sigma) and incubated for 2 h, 4°C with rotation. The immunoprecipitated proteins were then washed with 100 volumes of CSK buffer before elution of the bound material by addition of 300 µg/µl 3×FLAG peptide (Sigma) in 20 µl of CSK buffer. The input (S1) and eluted fraction were analyzed by Western blotting using antibodies as described above except for CID, which was detected with affinity purified rabbit polyclonal antibodies; 50 µg of total input protein and 25% of immunoprecipitated material were used for Western blot analysis.

## Supporting Information

Figure S1 SNAP-CID supports normal centromere function in the absence of endogenous CID. Endogenous CID was knocked-down using RNAi against the 3′ UTR of the CID mRNA. A) Images of S2 cells and clonal S2 cells stably expressing SNAP-CID. Endogenous CID intensity (green) is reduced after transfection with RNAi targeted against the 3′ UTR of CID while SNAP-CID intensity (red) remains constant and the protein is correctly targeted to the centromere, similarly to mock-treated cells. IF with anti-tubulin (blue) reveals that while mock-treated cells and SNAP-CID cells are mitotically normal after RNAi, untransfected S2 cells display chromosome segregation defects (arrow). Bar 5 µm. B) Western blots to determine protein levels of CID versus SNAP-CID after RNAi against endogenous CID. CAL1 levels are unaffected by loss of endogenous CID. Lamin acts as a loading control.
(PDF)

Figure S2 Inheritance of CID, CAL1 and CENP-C through one cell division. A) Distribution of TMR-CID intensity values on Day 1 (red shading) and Day 2 (blue shading) shows that the average decrease in intensity is in agreement with intensity distributions for individual cells. Arrows point to the mean TMR CID intensity for Day 1 and Day 2. B) Representative images from cells visualized immediately after labeling with TMR (Day 1) and 24 h later (Day 2) show that TMR-labeled CENP-C and CAL1 intensities are reduced following one cell division. TMR-labeled CENP-C and CAL1 is shown in red and DAPI in grey. The total number of cells quantified is indicated (n). CAL1 intensity showed a more dramatic reduction than that of CENP-C following one cell division. Bar = 15 µm.
(PDF)

Figure S3 Distribution of TMR-CAL1 and TMR-CENP-C through one cell division. A) Distribution of TMR-CENP-C intensity values on Day 1 (red shading) and Day 2 (blue shading) shows that the average decrease in intensity agrees with the intensity distributions for individual cells. Arrows point to the mean TMR CENP-C intensity for Day 1 and Day 2. B) Distribution of TMR-CAL1 intensity values as described for (A).
(PDF)

Figure S4 Mitotic loading of SNAP-CID. A) Images of BTP-blocked SNAP-CID cells. TMR is shown in red, total CID in green, phospho H3 in blue, and DAPI in grey. Bar = 15 µm. The efficiency of the blocking was consistently above 97%. B) FACS profiles showing the DNA content of S2 SNAP-CID cells from the quench-chase-pulse experiments in Figure 2. Samples for FACS were taken at 1, 2, 10, and 24 h following addition of BTP-block. The FACS profiles show that the distributions of cells in G1, S, and G2 are consistent between all time points. The approximate percentages of cells in each stage were estimated using FlowJo software (Tree Star, Inc.). C) Graphs showing the percent of cells containing new SNAP-CID (y-axis) in interphase versus mitosis at 1, 2, 10, 24 h. Bars indicate standard error. *** p<0.0001 (Fisher's exact test; n>100 cells per time-point).
(PDF)

Figure S5 New CID recruitment in SNAP-Kc167 cells. CID is recruited in mitosis in the independent Drosophila cell type, Kc167. Percent of interphase and mitotic cells showing new SNAP-CID recruitment at 0, 1, 2, 10, and 24 h following BTP-block in clonal SNAP-CID Kc167 cells. New CID recruitment is visible within the first hour following BTP-block and incorporation occurs preferentially in mitotic cells (>50% of mitotic cells are TMR-CID positive at 1 h). Bars indicate standard error. *** p<0.0001 (Fisher's exact test; n>100 cells per time-point).
(PDF)

Figure S6 Quantification of new CID loading in arrested interphase cells and SNAP-CID loading visualized 1 h after BTP-block. A) Representative images of interphase and mitotic SNAP-CID cells that were TMR labeled immediately after the BTP block (left panel). In both groups, 91% of cells were negative for any TMR-CID at the centromere. In the right panel, representative images of cells scored as positive for TMR-CID showing the clear difference in intensity of signal between mitotic (84% positive; n = 60) and interphase (36% positive; n = 230) cells, which show much weaker TMR-CID signal. ***p-value<0.0001 for mitotic versus interphase positive cells (Fisher's exact test). B) Representative images showing recruitment of TMR-CID (red) 1 h after the BTP-block. Centromeric TMR-CID is rarely observed in interphase (Inter), but it is visible in metaphase (Meta), anaphase/telophase (Ana/Telo) and cytokinesis/G1 (Cyto/G1). IF with anti-tubulin (green), anti-phospho H3 (gray), and DAPI (blue) was used to identify specific mitotic stages. Bar = 5 µm.
(PDF)

Figure S7 Mitotic loading of SNAP-CAL1. Graphs showing the percent of cells containing new SNAP-CAL1 (y-axis) in interphase versus mitosis at 1, 2, 10, 24 h. Bars indicate standard error. *** p<0.0001 (Fisher's exact test; n>100 cells per time-point).
(PDF)

Figure S8 New CENP-C is replenished during both mitosis and interphase in S2 cells. A) Percent of interphase and mitotic cells showing recruitment of new SNAP-CENP-C 10 h following the BTP block. New SNAP-CENP-C is equally detectable in both interphase (47%) and mitosis (48%), (p = 1 Fisher's exact test). B) Representative image showing new SNAP-CENP-C recruitment in a mitotic cell in late anaphase/telophase (lower left), and an interphase cell (upper right). TMR-labeled CENP-C is shown in red, GFP-tubulin in green, phospho H3 in gray, and DAPI in blue. Bar  = 5 µm.
(PDF)
