# Supplementary figures and images for: Assembly of Drosophila Centromeric Chromatin Proteins during Mitosis
Source: PLoS Genet. 2011 May 12;7(5):e1002068. doi: 10.1371/journal.pgen.1002068 (PMC3093364; doi:10.1371/journal.pgen.1002068)

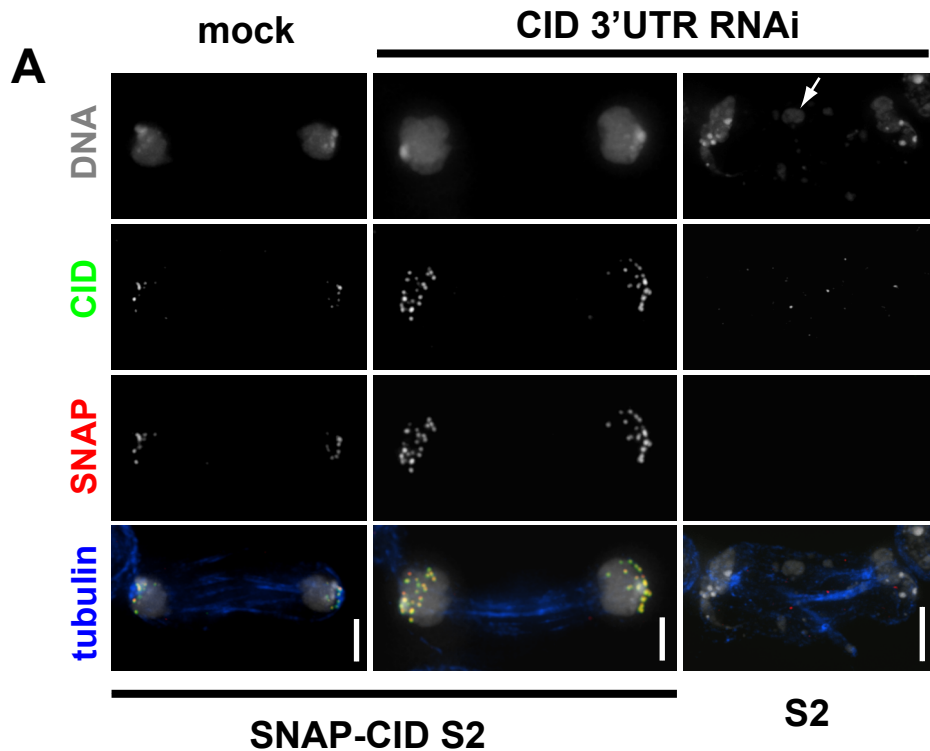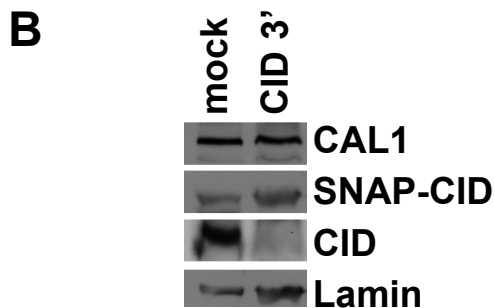

**Figure S1**

Supplement: Figure S1 — SNAP-CID supports normal centromere function in the absence of endogenous CID. Endogenous CID was knocked-down using RNAi against the 3′ UTR of the CID mRNA. A) Images of S2 cells and clonal S2 cells stably expressing SNAP-CID. Endogenous CID intensity (green) is reduced after transfection with RNAi targeted against the 3′ UTR of CID while SNAP-CID intensity (red) remains constant and the protein is correctly targeted to the centromere, similarly to mock-treated cells. IF with anti-tubulin (blue) reveals that while mock-treated cells and SNAP-CID cells are mitotically normal after RNAi, untransfected S2 cells display chromosome segregation defects (arrow). Bar 5 µm. B) Western blots to determine protein levels of CID versus SNAP-CID after RNAi against endogenous CID. CAL1 levels are unaffected by loss of endogenous CID. Lamin acts as a loading control. (PDF) [file pgen.1002068.s001.pdf]

Figure S2

A

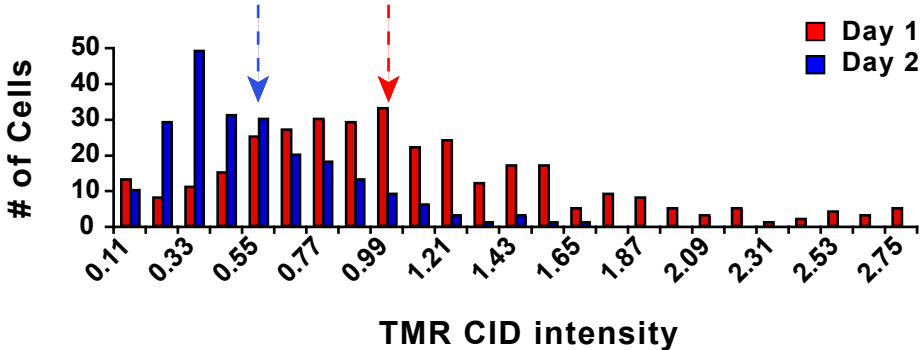

B

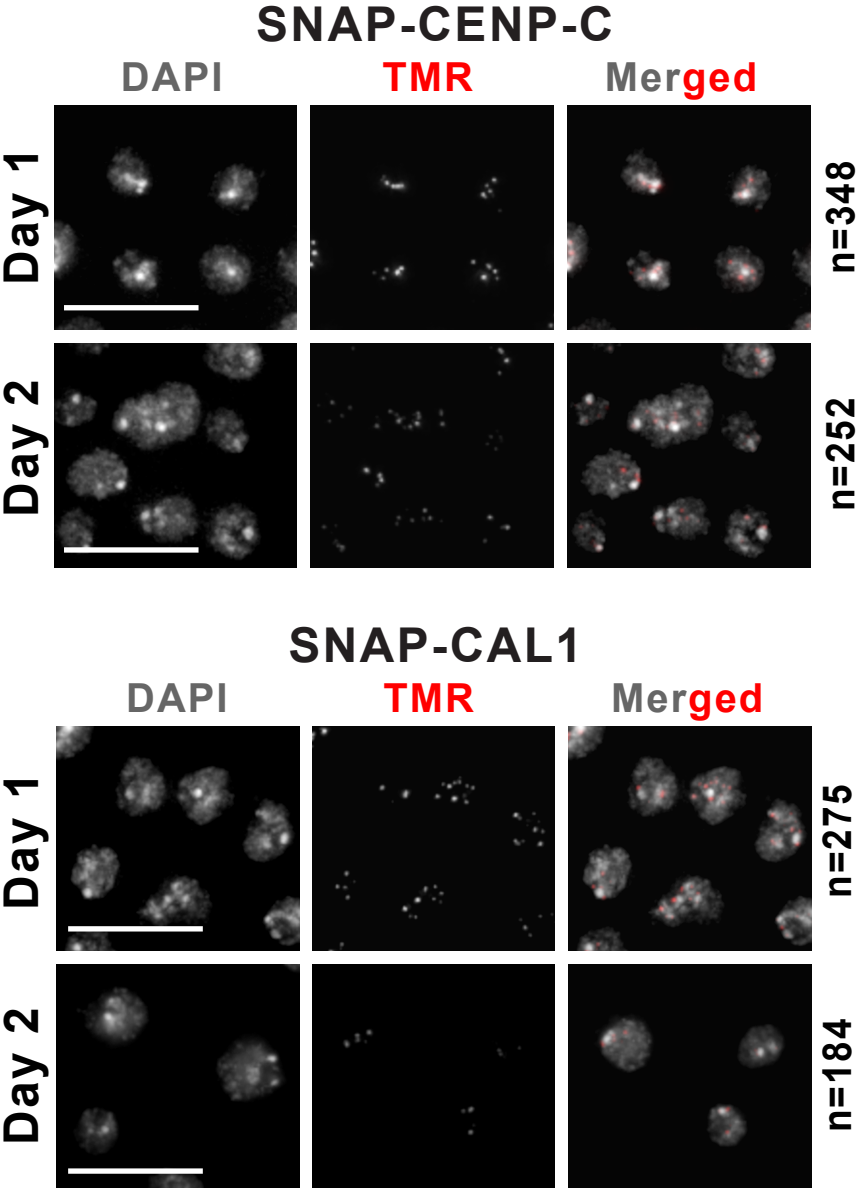

Supplement: Figure S2 — Inheritance of CID, CAL1 and CENP-C through one cell division. A) Distribution of TMR-CID intensity values on Day 1 (red shading) and Day 2 (blue shading) shows that the average decrease in intensity is in agreement with intensity distributions for individual cells. Arrows point to the mean TMR CID intensity for Day 1 and Day 2. B) Representative images from cells visualized immediately after labeling with TMR (Day 1) and 24 h later (Day 2) show that TMR-labeled CENP-C and CAL1 intensities are reduced following one cell division. TMR-labeled CENP-C and CAL1 is shown in red and DAPI in grey. The total number of cells quantified is indicated (n). CAL1 intensity showed a more dramatic reduction than that of CENP-C following one cell division. Bar = 15 µm. (PDF) [file pgen.1002068.s002.pdf]

# Figure S3

## A

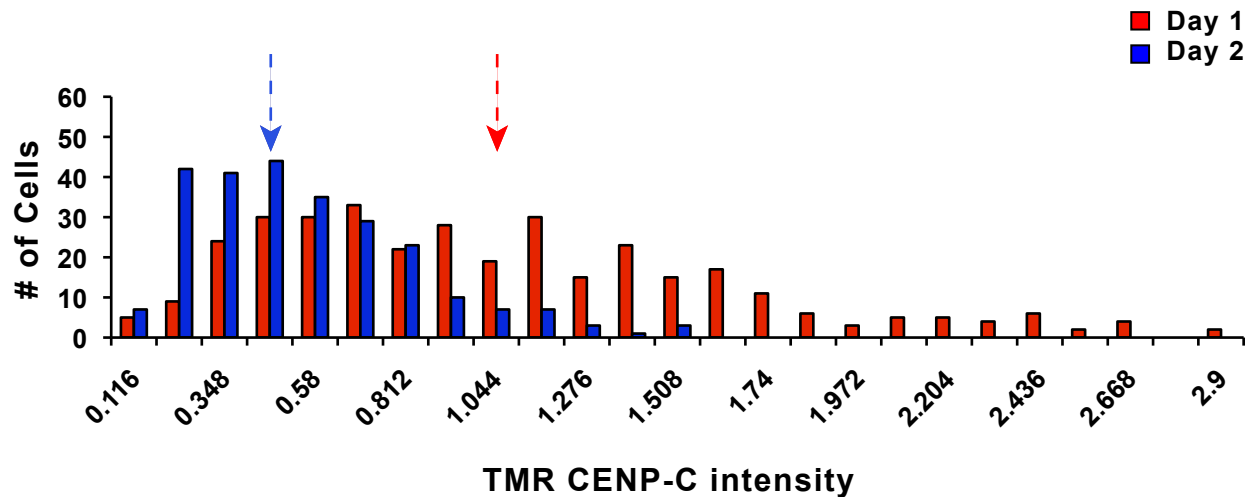

## B

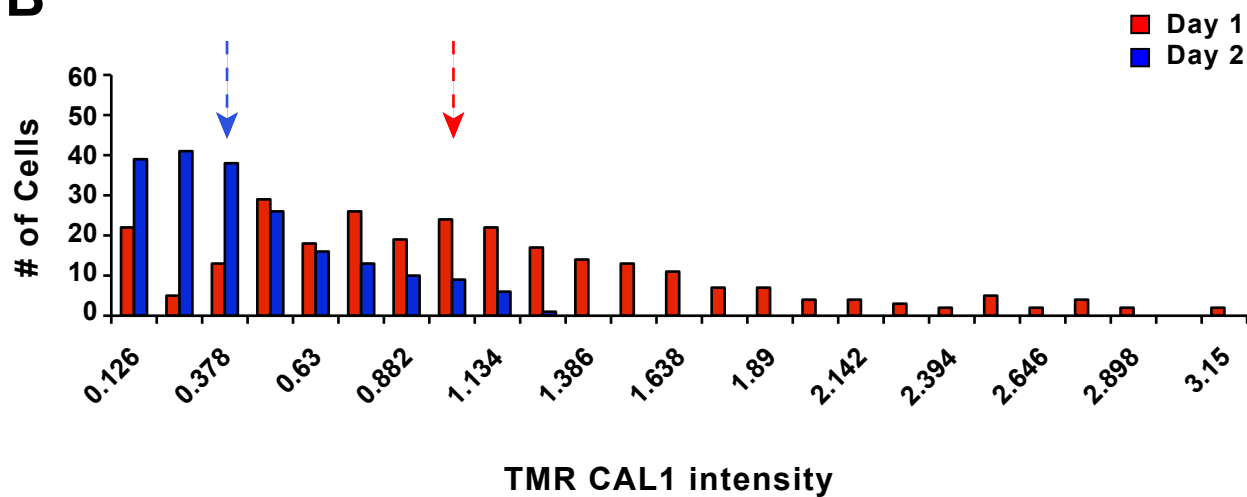

Supplement: Figure S3 — Distribution of TMR-CAL1 and TMR-CENP-C through one cell division. A) Distribution of TMR-CENP-C intensity values on Day 1 (red shading) and Day 2 (blue shading) shows that the average decrease in intensity agrees with the intensity distributions for individual cells. Arrows point to the mean TMR CENP-C intensity for Day 1 and Day 2. B) Distribution of TMR-CAL1 intensity values as described for (A). (PDF) [file pgen.1002068.s003.pdf]

A

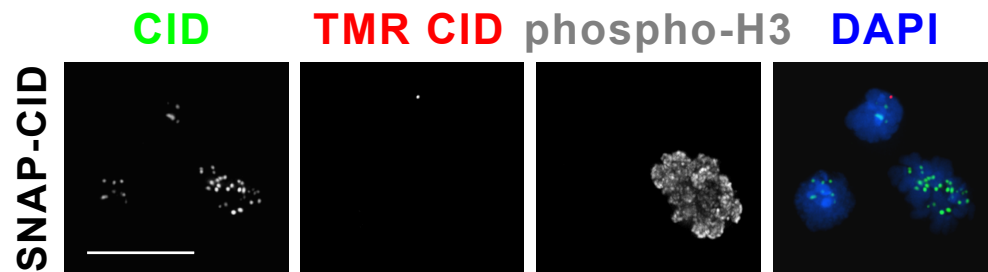

B

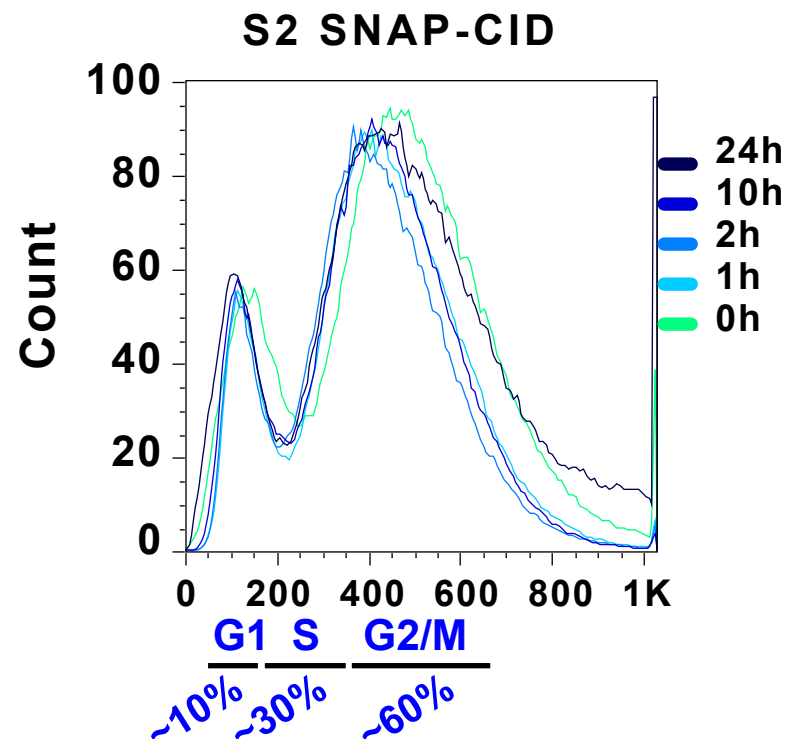

C

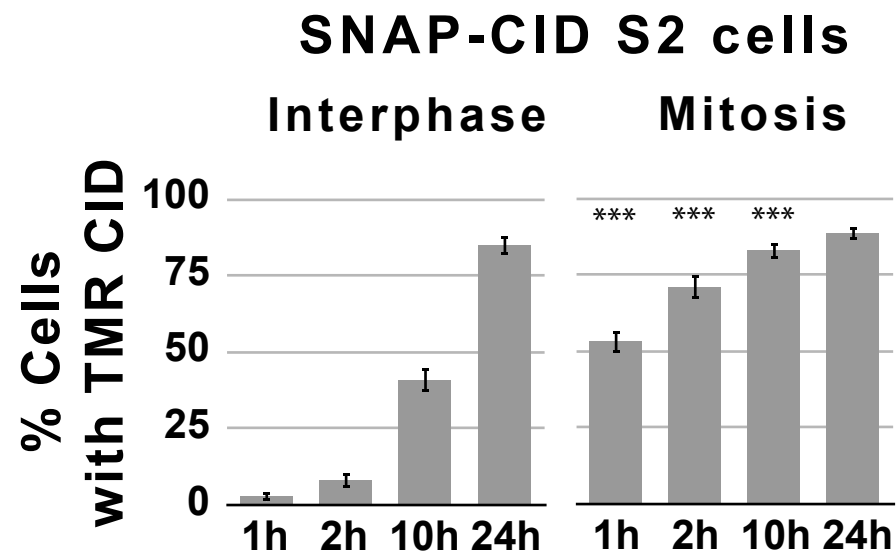

Figure S4

Supplement: Figure S4 — Mitotic loading of SNAP-CID. A) Images of BTP-blocked SNAP-CID cells. TMR is shown in red, total CID in green, phospho H3 in blue, and DAPI in grey. Bar = 15 µm. The efficiency of the blocking was consistently above 97%. B) FACS profiles showing the DNA content of S2 SNAP-CID cells from the quench-chase-pulse experiments in Figure 2. Samples for FACS were taken at 1, 2, 10, and 24 h following addition of BTP-block. The FACS profiles show that the distributions of cells in G1, S, and G2 are consistent between all time points. The approximate percentages of cells in each stage were estimated using FlowJo software (Tree Star, Inc.). C) Graphs showing the percent of cells containing new SNAP-CID (y-axis) in interphase versus mitosis at 1, 2, 10, 24 h. Bars indicate standard error. *** p<0.0001 (Fisher's exact test; n>100 cells per time-point). (PDF) [file pgen.1002068.s004.pdf]

## SNAP-CID Kc cells

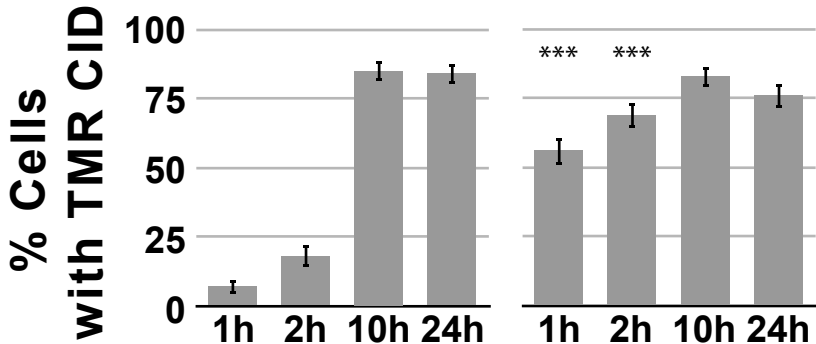

**Figure S5**

Supplement: Figure S5 — New CID recruitment in SNAP-Kc167 cells. CID is recruited in mitosis in the independent Drosophila cell type, Kc167. Percent of interphase and mitotic cells showing new SNAP-CID recruitment at 0, 1, 2, 10, and 24 h following BTP-block in clonal SNAP-CID Kc167 cells. New CID recruitment is visible within the first hour following BTP-block and incorporation occurs preferentially in mitotic cells (>50% of mitotic cells are TMR-CID positive at 1 h). Bars indicate standard error. *** p<0.0001 (Fisher's exact test; n>100 cells per time-point). (PDF) [file pgen.1002068.s005.pdf]

**A**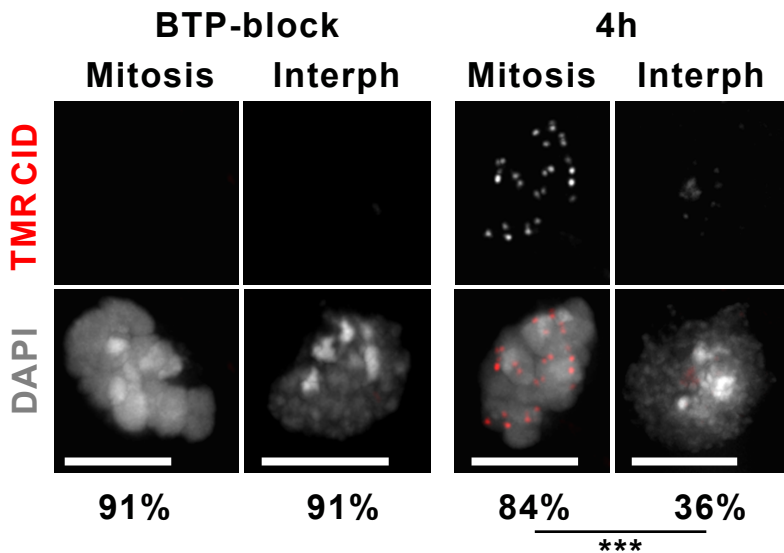**B**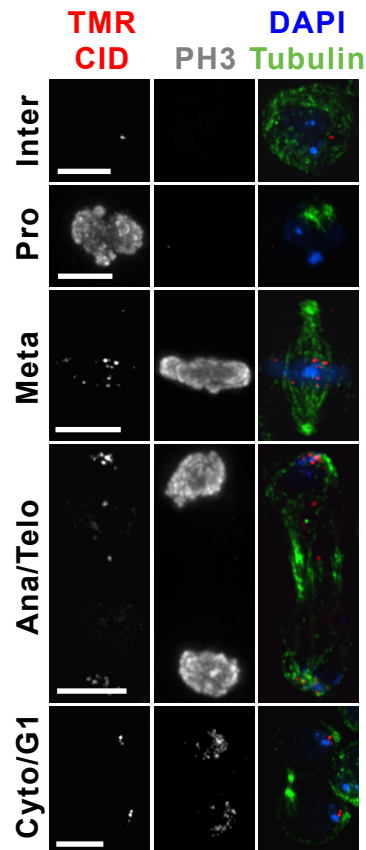**Figure S6**

Supplement: Figure S6 — Quantification of new CID loading in arrested interphase cells and SNAP-CID loading visualized 1 h after BTP-block. A) Representative images of interphase and mitotic SNAP-CID cells that were TMR labeled immediately after the BTP block (left panel). In both groups, 91% of cells were negative for any TMR-CID at the centromere. In the right panel, representative images of cells scored as positive for TMR-CID showing the clear difference in intensity of signal between mitotic (84% positive; n = 60) and interphase (36% positive; n = 230) cells, which show much weaker TMR-CID signal. ***p-value<0.0001 for mitotic versus interphase positive cells (Fisher's exact test). B) Representative images showing recruitment of TMR-CID (red) 1 h after the BTP-block. Centromeric TMR-CID is rarely observed in interphase (Inter), but it is visible in metaphase (Meta), anaphase/telophase (Ana/Telo) and cytokinesis/G1 (Cyto/G1). IF with anti-tubulin (green), anti-phospho H3 (gray), and DAPI (blue) was used to identify specific mitotic stages. Bar = 5 µm. (PDF) [file pgen.1002068.s006.pdf]

## SNAP-CAL1 S2 cells

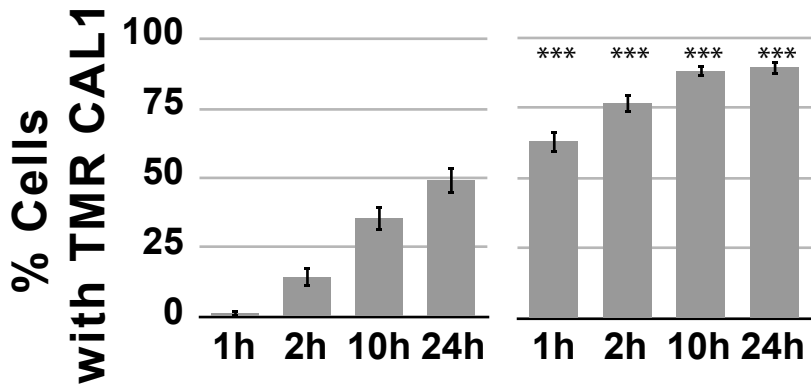

**Figure S7**

Supplement: Figure S7 — Mitotic loading of SNAP-CAL1. Graphs showing the percent of cells containing new SNAP-CAL1 (y-axis) in interphase versus mitosis at 1, 2, 10, 24 h. Bars indicate standard error. *** p<0.0001 (Fisher's exact test; n>100 cells per time-point). (PDF) [file pgen.1002068.s007.pdf]

**A**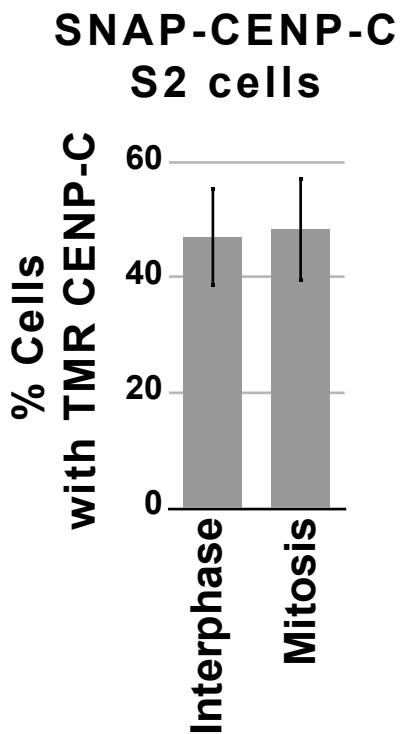**B**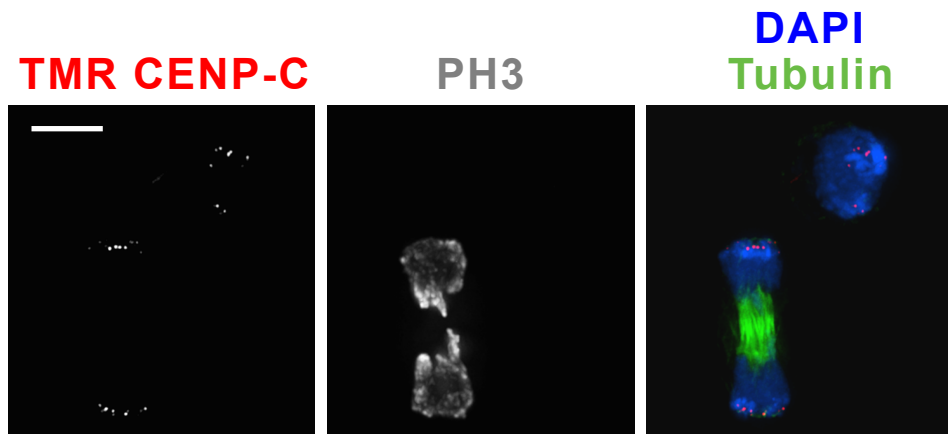**Figure S8**

Supplement: Figure S8 — New CENP-C is replenished during both mitosis and interphase in S2 cells. A) Percent of interphase and mitotic cells showing recruitment of new SNAP-CENP-C 10 h following the BTP block. New SNAP-CENP-C is equally detectable in both interphase (47%) and mitosis (48%), (p = 1 Fisher's exact test). B) Representative image showing new SNAP-CENP-C recruitment in a mitotic cell in late anaphase/telophase (lower left), and an interphase cell (upper right). TMR-labeled CENP-C is shown in red, GFP-tubulin in green, phospho H3 in gray, and DAPI in blue. Bar = 5 µm. (PDF) [file pgen.1002068.s008.pdf]
